# Joint estimation of hand-foot-mouth disease model and prediction in korea using the ensemble kalman filter

**DOI:** 10.1371/journal.pcbi.1012996

**Published:** 2025-04-17

**Authors:** Wasim Abbas, Sieun Lee, Sangil Kim

**Affiliations:** 1 Nonlinear Dynamics and Mathematical Application Center, Kyungpook National University, Daegu, Republic of Korea; 2 Innovation Center for MathScience Research & Education, Pusan National University, Busan, Republic of Korea; 3 Department of Mathematics, Pusan National University, Busan, Republic of Korea; 4 Institute for Future Earth, Pusan National University, Busan, Republic of Korea; Los Alamos National Laboratory, UNITED STATES OF AMERICA

## Abstract

**Background:**

In Korea, Hand-foot-and-mouth disease (HFMD) is a recurring illness that presents significant public health challenges, primarily because of its unpredictable epidemic patterns. The accurate prediction of the spread of HFMD plays a vital role in the effective management of the disease.

**Methods:**

We have devised a dynamic model that accurately represents the transmission dynamics of HFMD. The model includes compartments for susceptible, exposed, inpatients, outpatients, recovered, and deceased individuals. By utilizing monthly inpatient and outpatient data, the ensemble Kalman filter (EnKF) method was employed to perform a joint estimation of model parameters and state variables. The calibration of model parameters involved using data from the months of January to May, while generating forecasts for the timeframe spanning from June to December.

**Results:**

The findings reveal a significant alignment between the model and the observed data, as evidenced by root-mean-square error (RMSE) values below 1000 for inpatients and below 10000 for outpatients starting in June. The correlation coefficients surpassed 0.9, except for the year 2015. The implications of our findings suggest a notable shift in transmission and recovery rates, starting in 2015.

**Discussion:**

The model successfully predicted the peak and magnitude of HFMD outbreaks occurring between June and December, closely matching the observed epidemic patterns. The model’s efficacy in predicting epidemic trends and informing preventive strategies is reinforced by the insights gained from monthly variations in parameter estimates of HFMD transmission dynamics.

## Summary

The cyclical nature of HFMD outbreaks in South Korea presents a significant and ongoing public health challenge, complicating both prevention and treatment strategies. Our study aimed to enhance forecasting accuracy for HFMD by developing a dynamic model that integrates real-time data using EnKF. Key disease dynamic factors, including susceptible, exposed, inpatient, outpatient, recovered, and deceased populations, are accounted for in this model. Employing monthly inpatient and outpatient data spanning 2011–2019, we conducted joint estimation of model parameters and states to forecast epidemic trends. The results of our study reveal a strong correspondence between predicted and actual HFMD trends, achieving a high degree of forecasting accuracy from June. The model precisely pinpointed peak epidemic periods and their severity, thus facilitating prompt and efficacious public health interventions. This research shows the utility of real-time data assimilation in improving the accuracy of infectious disease predictions, informing mitigation strategies for HFMD on a global scale, including South Korea. By bridging epidemiological modeling with actionable forecasts, our work contributes to advancing public health preparedness and reducing HFMD-related morbidity and mortality.

## Background

The contagious and predominantly pediatric nature of HFMD presents a substantial public health challenge on a global scale. The etiology of the illness is attributed to viral infections in the intestines, primarily Coxsackievirus A16 (CV-A16) and Enterovirus 71 (EV-71) [1]. Following exposure to these enteroviruses, viral replication occurs in the tissues beneath the mucous membrane of the throat or intestines, starting an incubation period of around 3–10 days [2]. Within this timeframe, the virus undergoes proliferation and subsequent migration to lymphatic tissue, ultimately resulting in symptomatic manifestations.

The symptoms of HFMD may differ based on which organs are impacted by the virus, resulting in varying clinical presentations. When the skin is affected, distinct blistering ulcers manifest on the hands, feet, and buttocks, frequently accompanied by fever. In cases of increased severity, HFMD can impact critical organs such as the brain and heart, leading to complications such as meningitis or myocarditis [3]. The transmission of HFMD primarily occurs through contact with infected secretions, either directly or indirectly through contaminated surfaces, or through exposure to environments where infected individuals may be present. Although most cases involve children under the age of 10 [4], there are also occurrences of adult infection [5].

The occurrence of HFMD has been predominantly observed in the western Pacific region since it was first discovered, with peak incidences during the seasons of spring, summer, and fall [2]. Countries like China have consistently reported tens of thousands of cases each year, with notable mortality rates recorded in certain years, particularly 2009 and 2010 [6]. The occurrence of HFMD has also been recorded in various other regions, such as Malaysia, Hungary, Bulgaria, and Spain, exhibiting an increasing pattern [7–9]. Since 2011, cases of HFMD have been consistently monitored in South Korea. Typically, the number of reported cases rises from May onwards, peaks in June, and remains prevalent throughout the summer and fall seasons [10]. To enhance monitoring and surveillance endeavors, the Korea Disease Control and Prevention Agency uses the Enterovirus Infectious Disease Pathogen Surveillance (KESS) system, classifying HFMD as a level 4 infectious disease.

The SEIQR model, widely used to study the transmission dynamics of HFMD, categorizes individuals into susceptible (S), exposed (E), infectious and not hospitalized (I), infectious and hospitalized (Q), and recovered (R) compartments [11]. For instance, the same study applied the minimum sum of squares (MSS) technique to estimate critical parameters such as transmission rate and recovery rate, affirming their pivotal role in shaping disease control strategies. Likewise, another research [12] focused on analyzing seasonal transmission patterns by employing the susceptible-exposed-infectious-asymptomatic-removed (SEIAR) model. Through the application of curve fitting methodologies, researchers can delineate the seasonality and transmissibility of HFMD, placing emphasis on the necessity of preemptive prevention policies prior to the occurrence of peak incidence periods [12].

Despite providing valuable insights into HFMD transmission dynamics, these studies could not effectively monitor real-time shifts in disease transmission because of heavy reliance on retrospective data analysis. Incorporating data assimilation techniques has emerged as a promising approach for real-time prediction in epidemiological models of infectious diseases [13–17].

The process of data assimilation (DA) integrates observational data with pre-existing scientific knowledge, such as mathematical models of physical processes. Integrating these components, the DA method aims to reconcile uncertainties inherent in observational data and model forecasts, yielding a more precise and dependable system representation [18]. In [13], the authors employed data assimilation to develop a prediction system that demonstrated remarkable accuracy in forecasting the spatiotemporal spread of influenza, including the onset week, peak week, and peak intensity. Similar initiatives have been pursued in the domain of HFMD research, with studies employing data assimilation methodologies, specifically EnKF, to estimate disease states in real-time [14]. The application of data assimilation in infectious disease models has typically been limited to state estimation, neglecting to fully capture the dynamic interaction of parameters that affect epidemic trajectories. To address these limitations, the study in [14] employs a joint estimation method that simultaneously estimates parameters and states within the framework of the susceptible-exposed-infectious-recovered (SEIR) model. Through the utilization of this method, the research aims to create an extensive forecasting system that can predict outbreaks of HFMD in real-time. This system will enable proactive intervention strategies and help reduce the impact of HFMD on public health.

The present study aims to address these challenges by developing a prediction system for HFMD spread in Korea by applying data assimilation methods. By simultaneously estimating parameters and states using data assimilation techniques, particularly the EnKF, the study seeks to provide timely and accurate forecasts of epidemic scales and peak timings. By analyzing HFMD inpatients and outpatients’ monthly data from 2011 to 2019, this study seeks to provide valuable insights for implementing proactive prevention strategies and enhancing readiness for upcoming outbreaks. This study is also using a more realistic model compared to [14] that incorporates both inpatients and outpatients’ data with differing transmission rates. The main goal of the study is to contribute to the reduction of HFMD-related illness and death, to protect public health in Korea and beyond.

## Methods

**Fig 1:**
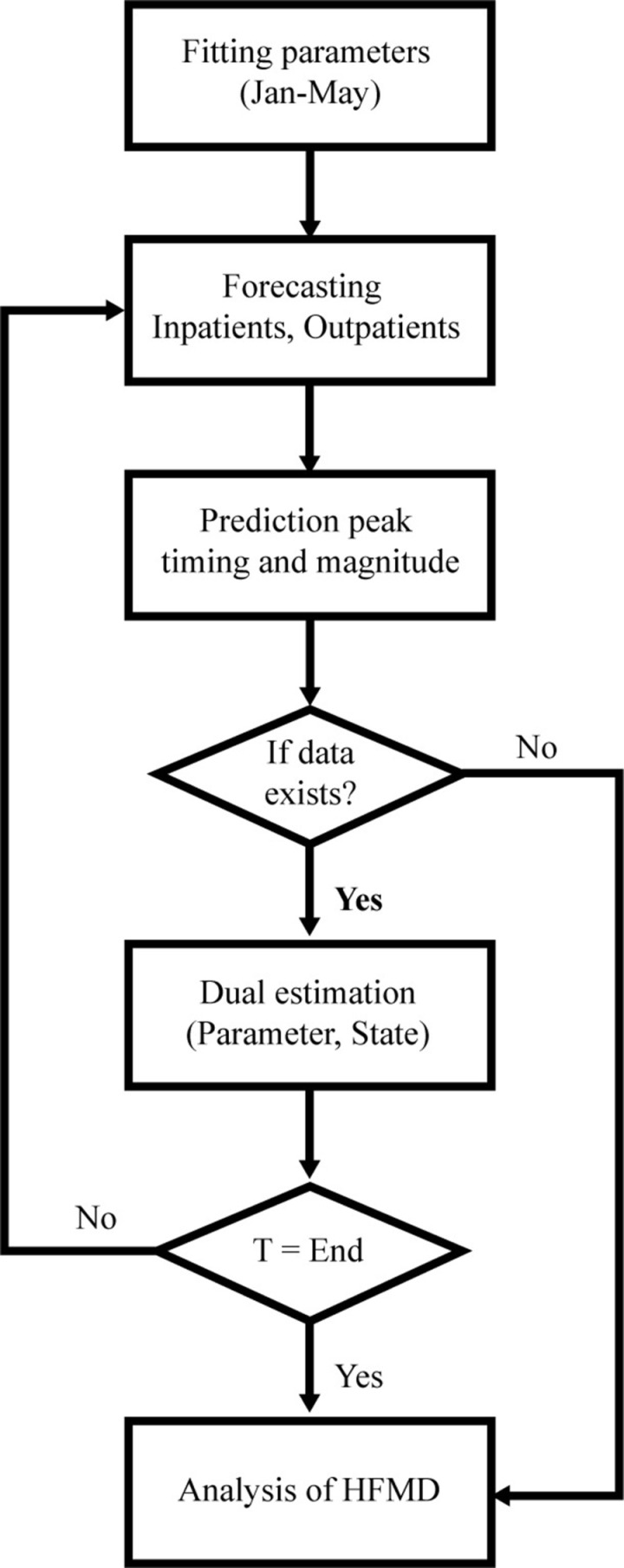
The framework of HFMD forecasting.

The delineation of HFMD forecasting and analysis, as depicted in Fig 1, encompasses three distinct stages: parameter fitting, forecasting, and analysis. The parameter fitting phase involves the utilization of monthly data sourced from inpatient and outpatient records provided by the National Health Review and Assessment Service [19].

Initially, data from January to May is used to calibrate model parameters through the application of EnKF method. In the subsequent forecasting stage, the estimated parameters are used to predict the remaining timeframe up to December. The process is then repeated by incrementally adding a month of data (e.g., January to June, January to July) and subsequently forecasting the remaining months. This iterative process continues until December, when all data for the year is fitted using EnKF. These projections offer significant insights into the expected peak and scale of the epidemic. In addition, EnKF assimilates the model and observational data for every temporal data point. This iterative refinement of parameters and states progressively enhances the forecasting process. In the analysis step, the parameters that vary monthly are carefully examined to identify temporal patterns in the dynamics of HFMD from 2011 to 2019.

## HFMD transmission model

The HFMD model represents a comprehensive framework for infectious diseases, encompassing both inpatients and outpatients. It classifies individuals into six distinct groups: susceptible (S), exposed (E), inpatients ($I_{in}$), outpatients ($I_{out}$), recovered (R) and death(D). The aggregate of all these groups represents the total population ($N_{pop}$).

In contrast to conventional SEIQR models, which integrate a quarantine compartment (Q) to denote isolated or hospitalized individuals, our model omits a distinct quarantine state. We delineate two infectious compartments—$I_{in}$ and $I_{out}$—to reflect the observed clinical heterogeneity of HFMD, considering severity and treatment requirements within inpatient and outpatient data from the National Health Review and Assessment Service [19].

In this model, $I_{in}$ represents inpatients—patients requiring extended care or observation within a healthcare facility, who are also at risk of disease-related mortality—while $I_{out}$ denotes outpatients—patients with milder symptoms receiving treatment without admission. These categories are established based on claims submitted for medical care benefits, with diagnoses assigned by healthcare providers according to patient symptoms, as documented in the data source [19]. This delineation differs from a quarantine compartment, which would typically include individuals isolated due to exposure or early symptoms prior to clinical classification. By focusing on $I_{in}$ and $I_{out}$, our framework reflects the post-diagnostic management of HFMD rather than preemptive isolation, aligning with the disease’s rapid progression and the data-driven approach outlined in Fig 1.

A set of equations that explain the intricate relationships among these compartments mathematically represented the model.


$$\frac{dS}{dt}=\mu N_{pop}+\gamma R-\frac{\beta \left(kI_{in}+I_{out}\right)S}{N_{pop}}-\mu S,$$

$$\frac{dE}{dt}=\frac{\beta \left(kI_{in}+I_{out}\right)S}{N_{pop}}-(\alpha +\mu )E,$$

$$\frac{dI_{in}}{dt}=\alpha \rho E-\left(\gamma_{1}+\mu +\delta \right)I_{in},$$

$$\frac{dI_{out}}{dt}=\alpha (1-\rho )E-\left(\gamma_{2}+\mu \right)I_{out},$$

$$\frac{dR}{dt}=\gamma_{1}I_{in}+\gamma_{2}I_{out}-(\mu +\gamma )R,$$

$$\frac{dD}{dt}=\delta I_{in}-\mu D,$$ (1)

where $N_{pop}=S+E+I_{in}+I_{out}+R+D$.

**Fig 2:**
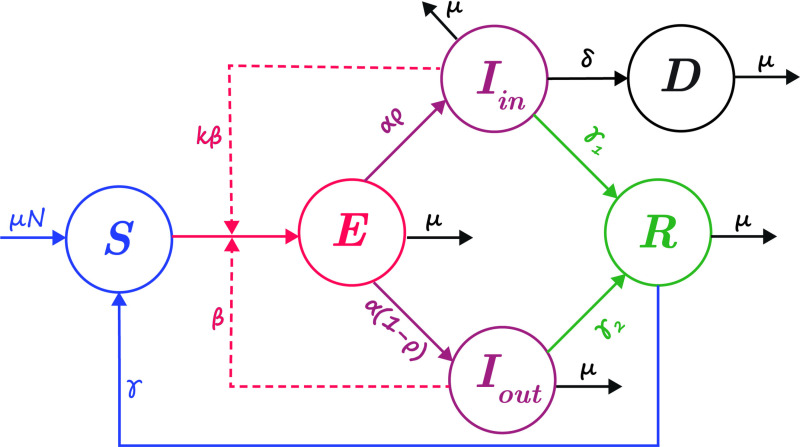
Flow chart of the HFMD model.

The flowchart that illustrates the HFMD model, as depicted in Fig 2, underscores eight pivotal parameters, each measured in monthly units. Within this set of parameters $\beta =\beta_{1}$ corresponds to the transmission rate originating from outpatients, whereas k signifies the factor of proportionality regulating the transmission rates between outpatients and inpatients, which is denoted as $\beta_{2}$ ($\beta_{2}=k\beta$). α represents the rate at which exposed individuals progress to the infectious stage, ρ denotes the fraction of exposed individuals who become inpatients, $\gamma_{1}$ signifies the recovery rate of inpatients, $\gamma_{2}$ represents the recovery rate of outpatients, while μ and δ denote the natural and disease related death rates, respectively.

## Stability analysis

We analyze the stability of the disease-free equilibrium (DFE) and endemic equilibrium (EE) for the system given by Equation (1). This analysis hinges on the basic reproduction number $R_{0}$, which determines the threshold behavior of the epidemic, and incorporates techniques such as [24,25].

### Boundedness

In our model (1), the total population $N_{pop}=S+E+I_{in}+I_{out}+R+D$ is constant, as $\frac{dN_{pop}}{dt}=\mu N_{pop}-\mu N_{pop}=0$, inherently bounding the system at $N_{pop}(t)=N_{pop}(0)$. To ensure biological feasibility, we prove solutions remain non-negative within this bound, following [24]. For initial conditions S(0), E(0), $I_{in}(0)$, $I_{out}(0)$, R(0), D(0)≥0 summing to $N_{pop}$, each compartment stays non-negative: if S=0, $\frac{dS}{dt}=\mu N_{pop}+\gamma R\geq 0$; if E=0, $\frac{dE}{dt}=\frac{\beta \left(kI_{in}+I_{out}\right)S}{N}\geq 0$; if $I_{in}=0$, $\frac{dI_{in}}{dt}=\alpha \rho E\geq 0$; if $I_{out}=0$, $\frac{dI_{out}}{dt}=\alpha (1-\rho )E\geq 0$; if R=0, $\frac{dR}{dt}=\gamma_{1}I_{in}+\gamma_{2}I_{out}\geq 0$; if D=0, $\frac{dD}{dt}=\delta I_{in}\geq 0$. Since N is fixed and each term is ≥0, it follows that $0\leq S,E,I_{in},I_{out},R,D\leq N_{pop}$, and the region $\text{\textbackslash{}\{}\left(S,E,I_{in},I_{out},R,D\right)|S,E,I_{in},I_{out},R,D\geq 0,S+E+I_{in}+I_{out}+R+D=N_{pop}\text{\textbackslash{}}\}$ is positively invariant, confirming boundedness.

Using the next-generation matrix method [26], we compute $R_{0}$ at the DFE $\left(S,E,I_{in},I_{out},R,D\right)=\left(N_{pop},0,0,0,0,0\right)$:


$$R_{0}=\frac{\beta }{\alpha +\mu }\left(\frac{k\alpha \rho }{\gamma_{1}+\mu +\delta }+\frac{\alpha (1-\rho )}{\gamma_{2}+\mu }\right)$$

### Disease-free equilibrium (DFE)

The DFE is given by:


$$\left(S,E,I_{in},I_{out},R,D\right)=\left(N_{pop},0,0,0,0,0\right)$$

#### Local stability.

The Jacobian at DFE yields eigenvalues, with stability determined by $R_{0}$ via the next-generation matrix method [27]. The DFE is locally asymptotically stable if $R_{0}<1$, and unstable if $R_{0}>1$.

#### Global stability.

Consider the Lyapunov function:


$$V=E+\frac{\beta k}{\gamma_{1}+\mu +\delta }I_{in}+\frac{\beta }{\gamma_{2}+\mu }I_{out}$$

Its derivative is:


$$\frac{dV}{dt}=\beta \left(kI_{in}+I_{out}\right)\left(\frac{S}{N_{pop}}-1\right)+(\alpha +\mu )\left(R_{0}-1\right)E$$

Since $\frac{S}{N_{pop}}-1\leq 0$ and $R_{0}-1<0$ when $R_{0}<1$, it follows that $\frac{dV}{dt}\leq 0$, ensuring global stability [28,29].

### Endemic equilibrium (EE)

The EE, existing when $R_{0}>1$, is:


$$S^{*}=\frac{N_{pop}}{R_{0}},\;E^{*}=\frac{N_{pop}\left(R_{0}-1\right)}{R_{0}B},\;I_{in}^{*}=\frac{\alpha \rho E^{*}}{\gamma_{1}+\mu +\delta },\;I_{out}^{*}=\frac{\alpha (1-\rho )E^{*}}{\gamma_{2}+\mu },$$

$$R^{*}=\frac{\gamma_{1}I_{in}^{*}+\gamma_{2}I_{out}^{*}}{\mu +\gamma },\;D^{*}=\frac{\delta I_{in}^{*}}{\mu },$$

here, $B=1+\frac{\alpha \rho }{\gamma_{1}+\mu +\rho }+\frac{\alpha (1-\rho )}{\gamma_{2}+\mu }+\frac{\alpha }{\gamma +\mu }\left(\frac{\gamma_{1}\rho }{\gamma 1+\mu +\delta }+\frac{\gamma_{2}(1-\rho )}{\gamma_{2}+\mu }\right)+\frac{\delta \alpha \rho }{\mu (\gamma 1+\mu +\delta )}$.

#### Local stability.

The EE is locally stable when $R_{0}>1$, as the DFE’s instability implies a stable EE via bifurcation [30].

#### Global stability.

Define the Lyapunov function:


$$V=\sum_{x\in \text{\textbackslash{}\{}S,E,I_{in},I_{out},R,D\text{\textbackslash{}}\}}x^{*}\left(\frac{x}{x^{*}}-1-\ln\frac{x}{x^{*}}\right)$$

Its derivative:


$$\frac{dV_{x}}{dt}=\left(1-\frac{x^{*}}{x}\right)\frac{dx}{dt}$$

- If $x>x^{*}$, then $\left(1-\frac{x^{*}}{x}\right)>0$ and $\frac{dx}{dt}<0$ (since x is moving towards $x^{*}$ therefore x is decreasing), so making the product negative.
- If $x<x^{*}$, then $\left(1-\frac{x^{*}}{x}\right)<0$ and $\frac{dx}{dt}>0$ (since x is moving towards $x^{*}$ therefore x is increasing), making the product negative.
- If $x=x^{*},$ then $\left(1-\frac{x^{*}}{x}\right)=0$ and $\frac{dx}{dt}=0$, making the product zero.

Thus, $\frac{dV}{dt}\leq 0$, with equality only at EE, proving global stability if $R_{0}>1$.

The DFE is globally stable if $R_{0}<1$, while EE is globally stable if $R_{0}>1$, highlighting the threshold role of $R_{0}$.

## Ensemble Kalman Filter for joint estimation

The Ensemble Kalman Filter (EnKF) was first developed by Evensen [31]. This method employs ensembles to approximate the probability density function (pdf) of the state by employing statistically representative values. These ensembles are used to calculate model covariance. The assimilation of the model’s state and observations is conducted under the principles of the Kalman filter [32]. By using the augmented EnKF method, it is possible to estimate both the state and unknown parameters of the model concurrently [33]. This requires the utilization of two models, namely a dynamical model and a measurement model, besides an augmented vector for the simultaneous estimation of parameters and states.

It is of utmost importance to emphasize that the ensemble Kalman filter does not explicitly compute the error covariance matrix $P_{k|k-1}\in R^{n\times n}$. Instead, it computes the matrices $P_{y}\in R^{m\times m}$ and $P_{xy}\in R^{n\times m}$, which correspond to $P_{k|k-1}C_{k}^{T}$ and $C_{k}P_{k|k-1}C_{k}^{T}+R_{k}$ as defined by the standard Kalman filter. For a more comprehensive analysis, please consult references [34] and [35].

The equations of relevance are:


$$x_{t+1}^{f}=M\left(t,x_{t}^{a}\text{, }\theta_{t}\right)+u_{t}\text{,}{\;u}_{t}\sim N\left(0\text{, }Q_{t}\right)$$ (2)

$$y_{t}=H\left(x_{t}^{f}\right)+v_{t}\text{, }v_{t}\sim N\left(0\text{, }R_{covt}\right)$$ (3)

$$s_{t+1}=\left[\begin{array}{c} x_{t+1}\\ \theta_{t+1} \end{array}\right]$$ (4)

Equation (1) represents a dynamic model. At time t, the state vector $x_{t}=\left[S_{t},\;E_{t},\;{I_{in}}_{t},\;{I_{out}}_{t},\;R_{t},\;D_{t}\right]$ is used to represent variables. We have two kinds of state vector: the prior state, denoted as $x_{t}^{f}$, and posterior state, denoted as $x_{t}^{a}$. M is the nonlinear operator for HFMD transmission model. The parameters vector is $\theta_{t}=[\beta_{t},\;k_{t},\;\gamma_{1_{t}},\gamma_{2_{t}},\;\rho_{t}]$. The model noise is $u_{t}$ that is assumed to zero mean normal distribution with covariance matrix $Q_{t}$. The equation (2) is measurement model. The observation vector is $y_{t}=[{I_{in}}_{t}\text{, }{I_{out}}_{t}]$, that contains numbers of inpatients and outpatients at each time t. The observation operator is H matrix which mapping the state value of dynamical model to observation (inpatients and outpatients). The $v_{t}$ is the measurement noise which assumed the zero mean normal distribution with covariance matrix $R_{covt}$. The vector $s_{t+1}$ is the augmented vector for using joint estimation.

This method comprises three steps: initialization, forecasting, and analysis steps. At the initialization steps, we set the initial distribution from the initial condition of dynamical model. In augmented EnKF, we choose not only the state values of the dynamical model but also parameters of the dynamical model. The initial augmented vector is $s_{0}=\left[\begin{array}{c} x_{0}^{f}\\ \theta_{0} \end{array}\right]=[s_{0}^{1},\;s_{0}^{2},\;\ldots ,\;s_{0}^{n}]$. The n is number of ensembles. We have the nth initial ensemble sets represented Initial condition pdf. The forecasting step is using the dynamical model that generates the prior. Then we get the $s_{t+1}^{f}=\left[\begin{array}{c} x_{t+1}^{f}\\ \theta_{t+1} \end{array}\right]=[s_{t+1}^{1},\;s_{t+1}^{2},\;\ldots ,\;s_{t+1}^{n}]$. The forecasting step repeats the simulation of the dynamical model until the measurements are met.

When measurement exists, the final step, the analysis step, is performed. For this purpose, perturbed observations are created using a measurement model. This is to prevent the ensemble covariance due to a single observation value from becoming very small [22].

The algorithm is:

1. Initialization: Generate an ensemble of size n for the initial state vectors:

$$s_{0}^{i}=\;s_{0}\text{, }i=1\text{, }2\text{, }3\text{, }\cdots \text{, }n$$ (5)

1. Prediction (Forecast step):
  - Forward each state vector realization for t=1, 2, 3, ⋯

$$x_{t+1}^{f,i}=M\left(t,x_{t}^{a},\theta_{t}\right)+u_{t}^{i}\text{, }i=1\text{, }2\text{, }3\text{, }\cdots \text{, }n$$ (6)

- Augmented state vector:

$$s_{t+1}^{f\text{, }i}=\left[\begin{array}{c} x_{t+1}^{f\text{, }i}\\ \theta_{t+1}^{i} \end{array}\right]$$

- Predicted (*a priori*) state estimate:

$$\overset{―}{s_{t+1}^{f}}=\frac{1}{n}\sum_{i=1}^{n}s_{t+1}^{f\text{, }i}$$

1. Update: Calculate predictions:
  - For each realization, compute the observation predictions:

$$y_{t+1}^{i}=H\left(x_{t+1}^{f,i}\right)\text{, }i=1\text{, }2\text{, }3\text{, }\cdots \text{, }n$$

- Calculate Ensemble mean of predictions:

$$\overset{―}{y_{t+1}}=\frac{1}{n}\sum_{i=1}^{n}y_{t+1}^{i}$$

1. Calculate covariance matrices:
  - **Cross-covariance** between the state and observations:

$$P_{sy_{t+1}}=\frac{1}{n-1}\sum_{i=1}^{n}\left(s_{t+1}^{f,i}-\overset{―}{s_{t+1}^{f}}\right){\left(y_{t+1}^{i}-\overset{―}{y_{t+1}}\right)}^{T}$$ (7)

- Predictive covariance of the observations:

$$P_{yy_{t+1}}=\frac{1}{n-1}\sum_{i=1}^{n}\left(y_{t+1}^{i}-\overset{―}{y_{t+1}}\right){\left(y_{t+1}^{i}-\overset{―}{y_{t+1}}\right)}^{T}$$ (8)

1. Calculate Kalman Gain:
  - Compute the Kalman Gain:

$$K_{t+1}=P_{sy_{t+1}}{\left(P_{yy_{t+1}}+R_{e_{t+1}}\right)}^{-1}$$ (9)

- Where, $R_{e_{t}}$ is the sample measurement noise covariance

$$R_{e_{t}}\;=\;\frac{1}{n-1}\sum_{i=1}^{n}\left(v_{t}^{i}\right){\left(v_{t}^{i}\right)}^{T}$$

1. Update (Analysis Step):
  - Update each state vector realization:

$$s_{t+1}^{a,i}=s_{t+1}^{f,i}+K_{t+1}\left({{\text{data}}_{t+1}+v_{t+1}^{i}-y}_{t+1}^{i}\right)\text{, }i=1\text{, }2\text{, }3\text{, }\cdots \text{, }n$$ (10)

- Update (*a posteriori*) state estimate

$$\overset{―}{s_{t+1}^{f}}=\frac{1}{n}\sum_{i=1}^{n}s_{t+1}^{f\text{, }i}$$

To optimize computational efficiency and accuracy, this study used an ensemble size of 1000 within the EnKF framework. Larger ensemble sizes mitigate sampling errors inherent in covariance estimation; an ensemble of 1000 members ensures reliable state and parameter estimation within our SEIQR model. This ensemble size is consistent with prior research, including meteorological forecasting studies, demonstrating stable performance with manageable computational demands [36].

## Genetic algorithm to find initial parameters and covariance matrices

The dynamical model was solved using the initial conditions $S_{0}=N_{pop}-\left(E_{0}+{I_{out}}_{0}+{I_{in}}_{0}\right)$, $E_{0}=N_{pop}\times 0.0005$ [14,37], $R_{0}=0$, and $D_{0}=0$. The initial values for ${I_{in}}_{0}$ and ${I_{out}}_{0}$ were obtained from available data [19]. Considering that the population of Korea is approximately 51 million [37], the population size $N_{pop}$ was set accordingly. To calibrate the parameters of the HFMD model, the initial parameters were selected. These parameters were sampled from a uniform distribution, with the following ranges: $\beta_{0}^{i}\sim U(0,1)$, $k_{0}^{i}\sim U(0,0.5)$, $\rho_{0}^{i}\sim U(0,0.04)$, ${\gamma_{1}}_{0}^{i}\sim U\left(\frac{1}{21},\frac{1}{6}\right)$, ${\gamma_{2}}_{0}^{i}\sim U\left(\frac{1}{21},\frac{1}{5}\right)$, and $\rho_{0}^{i}\sim U(0,\;0.05)$. Table 1 presents a summary of the estimated parameters, alongside those reported in other studies, offering a complete account of the calibration and parameter selection procedures.

**Table 1:** Description and value of parameters from reference and estimated by EnKF.

Symbols	Description	Value	Source
β	Transmission rate from outpatients	–	Table C in S1 File
k	Proportion of outpatient and inpatient transmission rates	–	Table C in S1 File
α	Rate of progression to the inpatients	$\frac{\text{1}}{\text{5}}\times \text{30}$	[2]
ρ	Proportion of the inpatients and outpatients	–	Table C in S1 File
γ	Proportion of loss of immunity	0.0202	[12,20]
$\gamma_{1}$	Recovery rate of inpatients	–	Table C in S1 File
$\gamma_{2}$	Recovery rate of outpatients	–	Table C in S1 File
μ	Natural birth/ death rate	$\frac{\text{5.44}}{\text{1000}\times \text{12}}$	[21]
δ	HFMD death rate	0.0003	[2,22,23]

Initially, the parameter values were estimated using MATLAB’s built-in genetic algorithm function (ga). These estimated parameters $\theta_{ga}$ with initial conditions were then utilized to solve the system of equations, resulting in estimated state $x_{ga}$. Here, Q is the covariance of $x_{ga}$ and $\theta_{ga}$, which accounts for uncertainty in both the state and parameter estimates.

The covariance matrix $R_{\text{cov}}$ is used to represent measurement noise. It is defined as $R_{\text{cov}}=\text{diag}\left[\sigma_{I_{in}}^{2},\sigma_{I_{out}}^{2}\right]$, where $\sigma_{I_{in}}$ and $\sigma_{I_{out}}$ signify the standard deviations of observation errors. The errors are set at a rate of ten percent of the observed data values for inpatient and outpatient cases, respectively.

## Error analysis

To measure the accuracy of the results of the fitting stage and forecasting stage, root mean square error (RMSE) and correlation were used. We define the predicted value as $\hat{y}$ the actual data value y and the accuracy measurement equation is as follows.


$$\text{RMSE}\left(y,\hat{y}\right)=\sqrt{\frac{1}{n}{\sum_{i=1}^{n}\left(y_{i}-\hat{y_{i}}\right)}^{2}}$$ (11)

$$\text{correlation}\left(y,\hat{y}\right)=\frac{\sum_{i=1}^{n}(y_{i}-\overset{―}{y_{i}\;})\left(\hat{y_{i}}-\overset{―}{\hat{y_{i}}}\right)\;}{\sqrt{\sum_{i=1}^{n}{\left(y_{i}-\overset{―}{y_{i}}\right)}^{2}}\sqrt{\sum_{i=1}^{n}(\hat{y_{i}}-\overset{―}{\hat{y_{i}}})}}$$ (12)

## Results

### Descriptive statistics

Utilizing National Health Insurance data from 2011 to 2019, covering the entirety of Korea’s population, the study relied on information provided by Korea’s National Health Review and Assessment Service [19] (see Tables A and B in S1 File). The data is reported monthly and was collected between January 1, 2011, and 2019. The code assigned to the HFMD data is ‘BO8.4’. Throughout the specified observation period, there is a consistent pattern of oscillations in both outpatients and inpatients.

**Fig 3:**
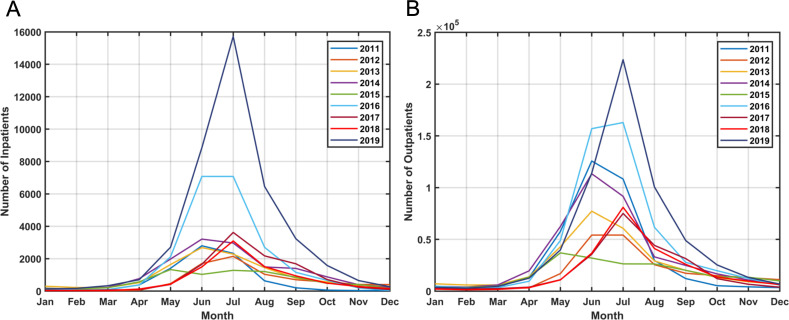
The HFMD data in Korea from 2011 to 2019. A) number of Inpatients, B) number of outpatients.

**Fig 4:**
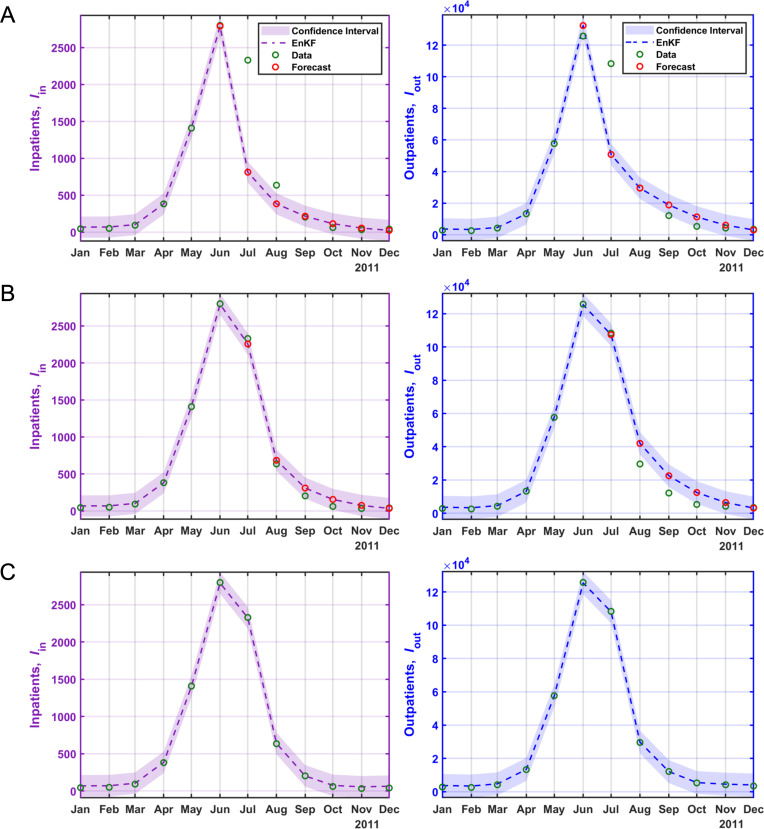
Fitting and forecasting results of HFMD inpatients and outpatients. Green circles represent inpatients and outpatients data. The red circles indicate the forecasts based on prior data, and the dashed lines depict the fit and forecast for inpatients and outpatients for the year 2011. **A)** Fitting the first 5 months and forecasting the next 7 months using prior data. **B)** Fitting the first 6 months and forecasting the next 6 months using prior data, **C)** fitting the entire dataset.

**Fig 5:**
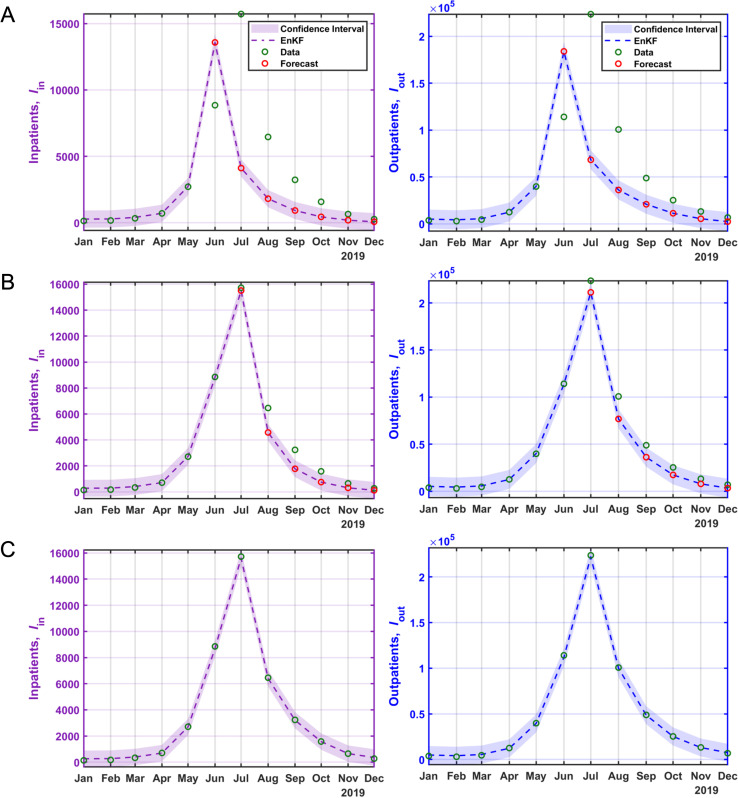
Fitting and forecasting results of HFMD inpatients and outpatients. Green circles represent inpatients and outpatients data. The red circles indicate the forecasts based on prior data, and the dashed lines depict the fit and forecast for inpatients and outpatients for the year 2019. **A)** Fitting the first 5 months and forecasting the next 7 months using prior data. **B)** Fitting the first 6 months and forecasting the next 6 months using prior data, **C)** fitting the entire dataset.

As a general trend, there is a steep rise in both outpatient and inpatient numbers from May onwards, peaking in July with the highest number of patients (see Fig 3). The prevalence of the epidemic varies across different years, with 2019 having the highest peak and 2015 having the lowest peak. The statistical data and model fitting for HFMD in the years 2011 and 2019 are visually represented in Figs 4 and 5. Refer to the S1 File (Figs E-K) for the model fitting and forecasting for the remaining years.

### Fitting results of HFMD inpatients and outpatients

The states (inpatients, outpatients) forecasting and fitting results of joint estimation using EnKF are shown in Figs 4 and 5. The results confirm that the peak timing of the rapidly increasing epidemic in inpatients and outpatients was well predicted, and the magnitude of the epidemic that varies every year was also predicted. These results show good fitting through EnKF’s joint parameter and state estimation.

### Estimation of transmission and recovery rate

**Fig 6:**
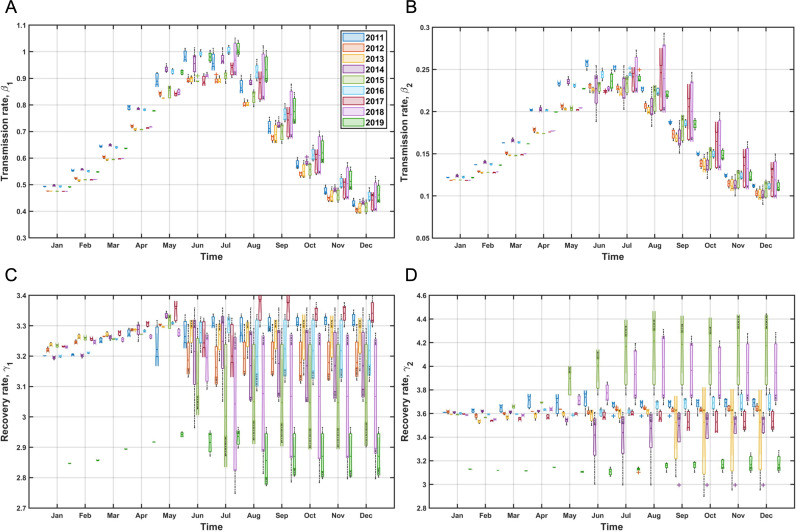
Estimation of the transmission and recovery rate during each month of the year 2011 to 2019. **A)** the transmission rate from inpatients $\left(\beta_{1}\right)$, **B)** the transmission rate from outpatients $\left(\beta_{2}\right)$, **C)** the recovery rate of inpatients $\left(\gamma_{1}\right)$ and **D)** the recovery rate of outpatients $\left(\gamma_{2}\right)$. The boxes represent the distribution of the ensemble of estimated parameters, with the center line representing the median and the edges of the box representing 25 percent and 75 percent.

The estimated values of transmission and recovery rate, crucial parameters of the HFMD model, are illustrated in Fig 6. From January to May is the data fitting stage and from June to December is the forecasting stage with estimated parameters.

Fig 6A) and C) depict the findings concerning inpatients. In all years, the transmission rate of inpatients exhibits a recurring pattern: it gradually rises from January, reaches its highest point in June or July, and then gradually declines. Each year, this pattern remains unchanged, but there has been a shift in magnitude since 2016. During the period from 2011 to 2015, the month with the highest value of *β* was June, whereas from 2016 to 2019, the highest value was observed in July.

Inpatients’ recovery rate shows a pattern of maintaining similar yearly values. When comparing the data, it becomes apparent that the year 2017 exhibits a unique pattern of fluctuations, with the highest values occurring in August and September. 2019 shows the lowest value among all years.

The results of Outpatients parameters are shown in Fig 6B), D). Like inpatients, the transmission rate of outpatients gradually increases from January, peaks in June or July, and then gradually decreases, repeating the same pattern. Unlike the case of inpatients, the pattern has changed since 2015. This change has the characteristic of maintaining values until August and then decreasing in same way as inpatients. The years that consistently maintain the highest values are 2011 and 2017. Changes in recovery rate can be divided into year groups where the pattern is maintained like that of inpatients and groups where it gradually increases. 2015 and 2018 have the highest values in all years, and the values also gradually increase. The year 2019 shows the lowest value, same as for inpatients. For further information one can see Figs A-D in S1 File.

The recovery rates for both inpatients and outpatients show variation, which expresses the dynamic nature of HFMD progression and the impact of factors like medical interventions, patient characteristics, and viral strain variations. Specifically, the recovery rates for inpatients (Fig 6 (C)) tend to be lower but more variable, likely reflecting the intensive medical interventions and varying treatment protocols applied in hospital settings. This variability suggests that inpatient recovery is influenced by the quality of healthcare services and the clinical management of severe cases.

For outpatients (Fig 6 (D)), the recovery rates exhibit a different pattern, with slightly higher but more stable values compared to inpatients. This consistency might be due to the standardized outpatient care practices and the self-limiting nature of milder HFMD cases.

### Forecasting accuracy of HFMD inpatients and outpatients

Forecasting inpatients and outpatients the accuracy between state and observations is evaluated through root-mean-squared-error and correlation. The values of RMSE and correlation accuracy can be found in Tables D-G in S1 File, respectively. The results of the accuracy evaluation are shown in Fig 5. The RMSE accuracy of Inpatients’ forecasting improves as the forecast start month increases, that is, as more observations are considered. It is confirmed that in all years, the accuracy is below 500 from the start time of June, with 2011 having the highest accuracy and 2019 having the lowest accuracy. The RMSE accuracy of outpatients’ forecasting is similar to the inpatients’ RMSE results, with high accuracy since June, with 2011 as the most accurate year, and the year with the lowest accuracy as 2015. The RMSE of the incidences rates is also a similar result, but the difference is that the accuracy is higher after July.

The result of the regression analysis indicates that the posterior analysis showed a superior fit to the observed data compared to the prior analysis, especially when considering a 10% observation error (see S1 File). Forecast accuracy for peak magnitudes improved with increased data availability (see Figs 4, 5 and E-K in S1 File).

## Discussion

HFMD is an annually recurring illness in Korea, with an increasing incidence trend prior to the COVID-19 pandemic. Owing to the multiple factors contributing to the epidemic, accurate forecasting of its pattern, scale, and timing poses a challenge. This study uses the $SEI_{in}I_{out}RD$ model [11] combined with EnKF for joint estimation of state and parameters using monthly inpatient and outpatient data (see Tables A and B in S1 File). The research involved parameter fitting from January to May and real-time forecasting from June to December, demonstrating the model’s robust performance in predicting the epidemic’s scale and peak.

Our study was conducted with the purpose of constructing a reliable forecasting model for HFMD in Korea. This was achieved by incorporating both inpatient and outpatient data and using EnKF to estimate both model parameters and state variables. Our model’s dynamic nature, featuring distinct compartments for inpatients and outpatients, offers a more detailed comprehension of HFMD transmission dynamics compared to prior research [14]. Our approach presents multiple advancements compared to this work. Zhan et al. [14] applied a singular dataset of incidence rates, whereas we integrated both inpatient and outpatient data, enabling a more comprehensive examination of HFMD transmission. The author’s model concentrated on a solitary infected category, solely estimating the general transmission and recovery rates. In contrast, our model differentiates between varying transmission rates among inpatients and outpatients, as well as distinct recovery rates for these groups, offering a more comprehensive comprehension of the dynamics of the disease.

In a similar vein, Baek et al. [38] performed an epidemiological and spatiotemporal examination of HFMD in Korea. However, they did not apply real-time forecasting or dynamic parameter estimation. Our research applies the technique of EnKF to update model parameters iteratively using real-time data, resulting in a substantial enhancement in the precision of our forecasts. Implementing this dynamic updating mechanism is essential to ensure timely and effective public health interventions.

**Fig 7:**
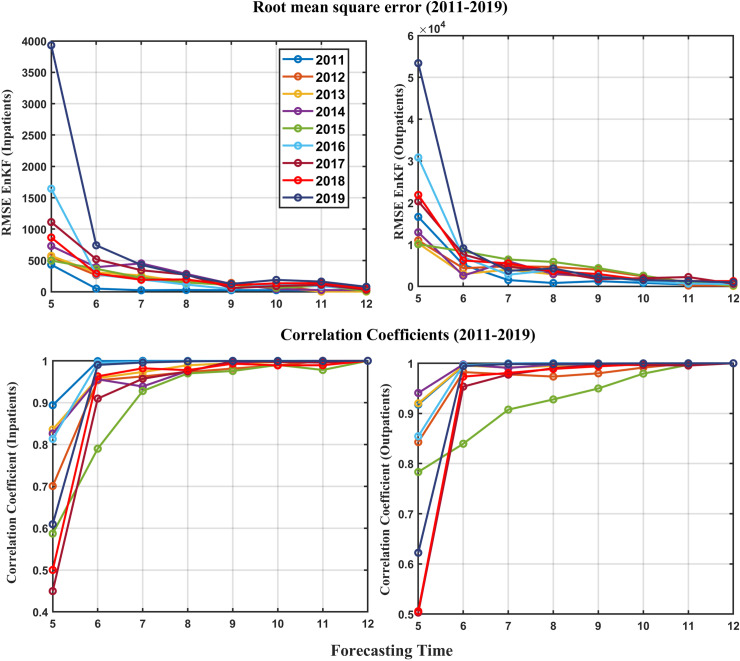
The forecast accuracy of HFMD inpatients and outpatients, from 2011 to 2019.

The forecasting accrual was confirmed by sequentially increasing data from May to December. Through this process, estimated inpatients and outpatients were computed to identify errors in monthly data and determine direct epidemic peaks. Correlation analysis was performed to assess the similarity of epidemic patterns. The EnKF technique yielded a high correlation coefficient (over 0.9) for most months (see Fig 7 and Tables F and G in S1 File), implying strong model accuracy, especially from June onwards. The RMSE values for inpatients were below 1000, and for the outpatients below 10000, further validating the model’s precision (see Fig 7, and Tables D and E in S1 File). From a forecasting standpoint, performance significantly improves beginning in June. In fact, HFMD frequently experiences a surge in occurrence during the months of June to August, which can be validated as a reliable predictive indicator. We had monthly variations in estimated parameters simultaneously with the state. Specifically, mirroring the observed trend of HFMD, the rate of transmission from both infections and outpatients exhibits a pattern that reaches its peak in June or July, following a gradual rise starting in January. When examining the data, a noticeable shift in patterns emerges for outpatients, suggesting a correlation with the rapid increase in patients from 2015 onwards.

The observed fluctuations in outpatient recovery rates could be influenced by external factors such as changes in public health policies, seasonal variations, and public awareness campaigns.

We have some limitations of research. (1) The use of monthly data limits the resolution of the model, potentially obscuring short-term variations and rapid changes in disease dynamics. (2) The model does not explicitly identify the direct causes of pattern changes, which are crucial for understanding and mitigating epidemic peaks. (3) The study focuses on short-term predictions. Long-term forecasting, incorporating multi-year parameter variations, is essential for developing comprehensive intervention strategies. (4) The model relies on several assumptions regarding transmission dynamics and parameter stability, which may not hold true under all circumstances. (5) Factors such as public health interventions, behavioral changes, and environmental conditions are not explicitly integrated into the model.

Despite these limitations, the joint estimation method employing EnKF presents a formidable tool for real-time forecasting and parameter estimation of HFMD. The application of this method can enhance our comprehension of HFMD transmission patterns and aid in creating efficient public health interventions. Future investigations should prioritize the refinement of data granularity, extension of the forecasting horizon, and incorporation of additional factors that influence the spread of diseases. These efforts will contribute to the improvement of model accuracy and applicability.

## Conclusion

In summary, our study presents an advanced model for forecasting HFMD in Korea. This model incorporates inpatient and outpatient data and employs the ensemble Kalman filter to estimate both model parameters and state variables simultaneously. This method presents notable enhancements compared to prior models. It achieves this by conducting a more comprehensive and dynamic analysis of the transmission dynamics of HFMD. HFMD outbreaks in Korea have been predicted with high accuracy by our model, which highlights its potential to inform public health strategies and reduce the impact of HFMD.

Integrating heterogeneous transmission rates and differentiated recovery rates for inpatients and outpatients in our model yields a comprehensive understanding of HFMD dynamics, a critical component in the development of precise intervention strategies. The EnKF method’s real-time updating mechanism guarantees the accuracy and relevance of our model, allowing for proactive public health responses.

Future investigations ought to concentrate on the improvement of the model by integrating additional data sources, such as environmental factors and population movement patterns, to augment the precision of predictions. The extension of our model’s implementation to diverse regions and diseases may yield valuable insights regarding the wider scope of our approach. Ultimately, our study aids in the ongoing initiatives to enhance epidemic forecasting and public health preparedness, with the target of mitigating the prevalence of HFMD and other infectious diseases.

## Supporting information

S1 FileTable A. Monthly inpatient data for Hand, Foot, and Mouth Disease (HFMD) from 2011 to 2019.Monthly counts of HFMD inpatients in Korea from 2011 to 2019, showing seasonal peaks between April and July and notable outbreaks in 2013, 2015, and 2019. **Table B. Monthly outpatient data for Hand, Foot, and Mouth Disease (HFMD) from 2011 to 2019.** Monthly counts of HFMD outpatient visits in Korea from 2011 to 2019, with seasonal peaks from May to July and significant surges in 2013, 2015, and 2019. **Table C. Mean values of estimated parameters using the ensemble Kalman filter.** Annual mean values of epidemiological parameters $\left(\beta ,k,\gamma_{1},\gamma_{2},\rho \right)$ estimated for HFMD in Korea from 2011 to 2019 using the ensemble Kalman filter. **Table D. Forecasting RMSE accuracy of HFMD inpatients from 2011 to 2019.** Root mean square error (RMSE) values for HFMD inpatient forecasts from 2011 to 2019, assessed for forecasting start months from May to December. **Table E. Forecasting RMSE accuracy of HFMD outpatients from 2011 to 2019.** RMSE values for HFMD outpatient forecasts from 2011 to 2019, evaluated for forecasting start months from May to December. **Table F. Forecasting correlation accuracy of HFMD inpatients from 2011 to 2019.** Correlation coefficients between observed and forecasted HFMD inpatient data from 2011 to 2019, calculated for forecasting start months from May to December. **Table G. Forecasting correlation accuracy of HFMD outpatients from 2011 to 2019.** Correlation coefficients between observed and forecasted HFMD outpatient data from 2011 to 2019, computed for forecasting start months from May to December. **Fig A. Change in parameter** $\beta_{1}$ **over the years 2011–2019.** Monthly variations in the transmission rate $\beta_{1}$ (from outpatient data) for HFMD in Korea from 2011 to 2019. **Fig B. Change in transmission rate** $\beta_{2}$ **observed from 2011 to 2019.** Monthly changes in the transmission rate $\beta_{2}$ (from inpatient data) for HFMD in Korea from 2011 to 2019. **Fig C. Change in recovery rate** $\gamma_{1}$ **observed from 2011 to 2019.** Monthly variations in the recovery rate $\gamma_{1}$ (for outpatients) for HFMD in Korea from 2011 to 2019. **Fig D. Change in recovery rate** $\gamma_{2}$ **observed from 2011 to 2019.** Monthly variations in the recovery rate $\gamma_{2}$ (for inpatients) for HFMD in Korea from 2011 to 2019. **Fig E. Real-time estimation and forecasting results of HFMD inpatients and outpatients in 2012.** Real-time estimation and forecasting of HFMD inpatients (brown circles) and outpatients (green circles) in 2012, with forecasts (red circles) and fitted trends (dashed lines). **Fig F. Real-time estimation and forecasting results of HFMD inpatients and outpatients in 2013.** Real-time estimation and forecasting of HFMD inpatients and outpatients in 2013, following the format of Fig E. **Fig G. Real-time estimation and forecasting results of HFMD inpatients and outpatients in 2014.** Real-time estimation and forecasting of HFMD inpatients and outpatients in 2014, consistent with Fig E. **Fig H. Real-time estimation and forecasting results of HFMD inpatients and outpatients in 2015.** Real-time estimation and forecasting of HFMD inpatients and outpatients in 2015, adhering to the style of Fig E. **Fig I. Real-time estimation and forecasting results of HFMD inpatients and outpatients in 2016.** Real-time estimation and forecasting of HFMD inpatients and outpatients in 2016, matching the format of Fig E. **Fig J. Real-time estimation and forecasting results of HFMD inpatients and outpatients in 2017.** Real-time estimation and forecasting of HFMD inpatients and outpatients in 2017, consistent with Fig E. **Fig K. Real-time estimation and forecasting results of HFMD inpatients and outpatients in 2018.** Real-time estimation and forecasting of HFMD inpatients and outpatients in 2018, following Fig E. **Fig L. Prior and posterior analysis of EnKF with different observation errors.** Correlation between observed HFMD data and prior forecasts (left panel) versus posterior analysis (right panel) under observation errors of 10%, 20%, 30%, 40%, and 50%, with coefficients and $R^{2}$ values.(DOCX)
