# Supplementary material for: Joint estimation of hand-foot-mouth disease model and prediction in korea using the ensemble kalman filter
Source: PLoS Comput Biol. 2025 Apr 17;21(4):e1012996. doi: 10.1371/journal.pcbi.1012996 (PMC12047828; doi:10.1371/journal.pcbi.1012996)
Supplement: S1 File — Monthly counts of HFMD inpatients in Korea from 2011 to 2019, showing seasonal peaks between April and July and notable outbreaks in 2013, 2015, and 2019. Table B. Monthly outpatient data for Hand, Foot, and Mouth Disease (HFMD) from 2011 to 2019. Monthly counts of HFMD outpatient visits in Korea from 2011 to 2019, with seasonal peaks from May to July and significant surges in 2013, 2015, and 2019. Table C. Mean values of estimated parameters using the ensemble Kalman filter. Annual mean values of epidemiological parameters (β,k,γ1,γ2,ρ) estimated for HFMD in Korea from 2011 to 2019 using the ensemble Kalman filter. Table D. Forecasting RMSE accuracy of HFMD inpatients from 2011 to 2019. Root mean square error (RMSE) values for HFMD inpatient forecasts from 2011 to 2019, assessed for forecasting start months from May to December. Table E. Forecasting RMSE accuracy of HFMD outpatients from 2011 to 2019. RMSE values for HFMD outpatient forecasts from 2011 to 2019, evaluated for forecasting start months from May to December. Table F. Forecasting correlation accuracy of HFMD inpatients from 2011 to 2019. Correlation coefficients between observed and forecasted HFMD inpatient data from 2011 to 2019, calculated for forecasting start months from May to December. Table G. Forecasting correlation accuracy of HFMD outpatients from 2011 to 2019. Correlation coefficients between observed and forecasted HFMD outpatient data from 2011 to 2019, computed for forecasting start months from May to December. Fig A. Change in parameter β1 over the years 2011–2019. Monthly variations in the transmission rate β1 (from outpatient data) for HFMD in Korea from 2011 to 2019. Fig B. Change in transmission rate β2 observed from 2011 to 2019. Monthly changes in the transmission rate β2 (from inpatient data) for HFMD in Korea from 2011 to 2019. Fig C. Change in recovery rate γ1 observed from 2011 to 2019. Monthly variations in the recovery rate γ1 (for outpatients) for HFMD in Korea from 2011 to [file pcbi.1012996.s001.docx]

**Supplementary File: Joint Estimation of Hand-Foot-Mouth Disease Model and Prediction in Korea Using the Ensemble Kalman Filter**

Wasim Abbas1, Sieun Lee2, Sangil Kim3,4*

**1** Nonlinear Dynamics and Mathematical Application Center, Kyungpook National University, Daegu, 41566, Republic of Korea

**2** Innovation Center for MathScience Research & Education, Pusan National University, Busan, 46241, Republic of Korea

**3** Department of Mathematics, Pusan National University, Busan, 46241, Republic of Korea

**4** Institute for Future Earth, Pusan National University, Busan, 46241, Republic of Korea

**Contents**

[**Table A.** **Monthly inpatient data for Hand, Foot, and Mouth Disease (HFMD) from 2011 to 2019.** Monthly counts of HFMD inpatients in Korea from 2011 to 2019, showing seasonal peaks between April and July and notable outbreaks in 2013, 2015, and 2019. 2](#_Toc194936782)

[**Table B.** **Monthly outpatient data for Hand, Foot, and Mouth Disease (HFMD) from 2011 to 2019.** Monthly counts of HFMD outpatient visits in Korea from 2011 to 2019, with seasonal peaks from May to July and significant surges in 2013, 2015, and 2019. 2](#_Toc194936783)

[**Table C. Mean values of estimated parameters using the ensemble Kalman filter.** Annual mean values of epidemiological parameters $\beta,k,\gamma1,\gamma2,\rho$ estimated for HFMD in Korea from 2011 to 2019 using the ensemble Kalman filter. 3](#_Toc194936784)

[**Table D. Forecasting RMSE accuracy of HFMD inpatients from 2011 to 2019.** Root mean square error (RMSE) values for HFMD inpatient forecasts from 2011 to 2019, assessed **for** forecasting start months from May to December. 3](#_Toc194936785)

[**Table E. Forecasting RMSE accuracy of HFMD outpatients from 2011 to 2019.** RMSE values for HFMD outpatient forecasts from 2011 to 2019, evaluated for forecasting start months from May to December. 3](#_Toc194936786)

[**Table F. Forecasting correlation accuracy of HFMD inpatients from 2011 to 2019.** Correlation coefficients between observed and forecasted HFMD inpatient data from 2011 to 2019, calculated for forecasting start months from May to December. 4](#_Toc194936787)

[**Table G. Forecasting correlation accuracy of HFMD outpatients from 2011 to 2019.** Correlation coefficients between observed and forecasted HFMD outpatient data from 2011 to 2019, computed for forecasting start months from May to December. 4](#_Toc194936788)

[**Fig A.** **Change in parameter** $\boldsymbol{\beta}\boldsymbol{1}$**​ over the years 2011 to 2019.** Monthly variations in the transmission rate $\beta1$​ (from outpatient data) for HFMD in Korea from 2011 to 2019. 5](#_Toc194936789)

[**Fig B. Change in transmission rate** $\boldsymbol{\beta}\boldsymbol{2}$ **observed from 2011 to 2019.** Monthly changes in the transmission rate $\beta2$​ (from inpatient data) for HFMD in Korea from 2011 to 2019. 6](#_Toc194936790)

[**Fig C. Change in recovery rate** $\boldsymbol{\gamma}\boldsymbol{1}$ **observed from 2011 to 2019.** Monthly variations in the recovery rate $\gamma1$​ (for outpatients) for HFMD in Korea from 2011 to 2019. 7](#_Toc194936791)

[**Fig D. Change in recovery rate** $\boldsymbol{\gamma}\boldsymbol{2}$ **observed from 2011 to 2019.** Monthly variations in the recovery rate $\gamma2$​ (for inpatients) for HFMD in Korea from 2011 to 2019. 8](#_Toc194936792)

[**Fig E. Real-time estimation and forecasting results of HFMD inpatients and outpatients in 2012.** Real-time estimation and forecasting of HFMD inpatients (brown circles) and outpatients (green circles) in 2012, with forecasts (red circles) and fitted trends (dashed lines). 9](#_Toc194936793)

[**Fig F. Real-time estimation and forecasting results of HFMD inpatients and outpatients in 2013.** Real-time estimation and forecasting of HFMD inpatients and outpatients in 2013, following the format of Fig. E. 10](#_Toc194936794)

[**Fig G. Real-time estimation and forecasting results of HFMD inpatients and outpatients in 2014.** Real-time estimation and forecasting of HFMD inpatients and outpatients in 2014, consistent with Fig. E. 11](#_Toc194936795)

[**Fig H. Real-time estimation and forecasting results of HFMD inpatients and outpatients in 2015.** Real-time estimation and forecasting of HFMD inpatients and outpatients in 2015, adhering to the style of Fig. E. 12](#_Toc194936796)

[**Fig I. Real-time estimation and forecasting results of HFMD inpatients and outpatients in 2016.** Real-time estimation and forecasting of HFMD inpatients and outpatients in 2016, matching the format of Fig. E. 13](#_Toc194936797)

[**Fig J. Real-time estimation and forecasting results of HFMD inpatients and outpatients in 2017.** Real-time estimation and forecasting of HFMD inpatients and outpatients in 2017, consistent with Fig. E. 14](#_Toc194936798)

[**Fig K. Real-time estimation and forecasting results of HFMD inpatients and outpatients in 2018.** Real-time estimation and forecasting of HFMD inpatients and outpatients in 2018, following Fig. E. 15](#_Toc194936799)

[**Fig L. Prior and posterior analysis of EnKF with different observation errors.** Correlation between observed HFMD data and prior forecasts (left panel) versus posterior analysis (right panel) under observation errors of 10%, 20%, 30%, 40%, and 50%, with coefficients and $R2$ values. 19](#_Toc194936800)

This supplementary file presents supplementary data and in-depth analyses that support the primary findings outlined in the study on employing the ensemble Kalman filter for the joint estimation of hand-foot-mouth disease prediction in Korea. It includes extensive estimations of transmission and recovery rates, real-time forecasting results, and accuracy assessments from 2011 to 2019. The purpose of the supplementary material is to present a thorough examination of the method and statistical evaluations performed, enhancing the comprehension of the model’s performance and its applicability in epidemiological forecasting. This supplementary file contains several figures and tables that provide additional support and validation for our findings. The figures and tables presented offer a comprehensive analysis of the fluctuations in crucial parameters, the precision of real-time predictions, and the efficacy of our joint estimation method over the period of estimation and forecasting.

## **Table A.** **Monthly inpatient data for Hand, Foot, and Mouth Disease (HFMD) from 2011 to 2019.** Monthly counts of HFMD inpatients in Korea from 2011 to 2019, showing seasonal peaks between April and July and notable outbreaks in 2013, 2015, and 2019.

| **Year** | **2011** | **2012** | **2013** | **2014** | **2015** | **2016** | **2017** | **2018** | **2019** |
| --- | --- | --- | --- | --- | --- | --- | --- | --- | --- |
| **Jan** | 45 | 32 | 301 | 184 | 171 | 137 | 45 | 33 | 137 |
| **Feb** | 50 | 30 | 222 | 112 | 125 | 63 | 46 | 29 | 170 |
| **Mar** | 93 | 22 | 219 | 231 | 210 | 82 | 66 | 30 | 333 |
| **Apr** | 382 | 54 | 524 | 768 | 570 | 376 | 93 | 127 | 704 |
| **May** | 1,409 | 462 | 1,640 | 1,979 | 1,329 | 2,116 | 414 | 428 | 2,711 |
| **Jun** | 2,799 | 1,704 | 2,684 | 3,210 | 1,032 | 7,079 | 1,654 | 1,494 | 8,853 |
| **Jul** | 2,329 | 2,145 | 2,281 | 2,969 | 1,283 | 7,078 | 3,621 | 3,095 | 15,721 |
| **Aug** | 635 | 1,037 | 1,446 | 1,490 | 1,207 | 2,705 | 2,173 | 1,511 | 6,461 |
| **Sep** | 203 | 701 | 803 | 1,401 | 854 | 1,157 | 1,686 | 941 | 3,232 |
| **Oct** | 61 | 558 | 567 | 876 | 479 | 664 | 712 | 492 | 1,580 |
| **Nov** | 33 | 411 | 368 | 393 | 411 | 200 | 242 | 283 | 651 |
| **Dec** | 41 | 405 | 295 | 226 | 162 | 51 | 96 | 154 | 270 |

## **Table B.** **Monthly outpatient data for Hand, Foot, and Mouth Disease (HFMD) from 2011 to 2019.** Monthly counts of HFMD outpatient visits in Korea from 2011 to 2019, with seasonal peaks from May to July and significant surges in 2013, 2015, and 2019.

| **Year** | **2011** | **2012** | **2013** | **2014** | **2015** | **2016** | **2017** | **2018** | **2019** |
| --- | --- | --- | --- | --- | --- | --- | --- | --- | --- |
| **Jan** | 2,821 | 2,690 | 7,095 | 4,724 | 4,201 | 4,878 | 2,758 | 1,990 | 3,814 |
| **Feb** | 2,552 | 2,414 | 5,959 | 3,994 | 3,680 | 3,357 | 2,013 | 1,393 | 2,974 |
| **Mar** | 4,177 | 2,332 | 5,952 | 5,970 | 4,841 | 3,422 | 2,455 | 1,729 | 4,588 |
| **Apr** | 13,277 | 3,221 | 13,810 | 19,602 | 13,588 | 9,562 | 3,850 | 3,399 | 12,550 |
| **May** | 57,662 | 16,950 | 42,884 | 62,446 | 36,983 | 48,999 | 10,994 | 10,882 | 39,811 |
| **Jun** | 125,738 | 54,148 | 77,292 | 113,401 | 31,961 | 156,992 | 35,636 | 36,741 | 114,079 |
| **Jul** | 108,310 | 54,167 | 60,676 | 91,636 | 26,324 | 162,920 | 75,113 | 81,003 | 223,582 |
| **Aug** | 29,597 | 25,558 | 29,158 | 33,113 | 26,249 | 61,693 | 44,196 | 41,170 | 100,667 |
| **Sep** | 12,135 | 17,243 | 20,075 | 24,858 | 20,140 | 27,804 | 31,461 | 25,892 | 48,852 |
| **Oct** | 5,269 | 14,982 | 13,260 | 16,676 | 13,754 | 19,724 | 12,048 | 13,605 | 25,305 |
| **Nov** | 4,175 | 13,021 | 9,001 | 10,716 | 13,464 | 12,275 | 6,659 | 9,701 | 13,248 |
| **Dec** | 3,421 | 11,105 | 6,856 | 7,011 | 9,895 | 5,818 | 3,840 | 6,372 | 6,838 |

## **Table C. Mean values of estimated parameters using the ensemble Kalman filter.** Annual mean values of epidemiological parameters $\left( \beta,k,\gamma_{1},\gamma_{2},\rho\right)$ estimated for HFMD in Korea from 2011 to 2019 using the ensemble Kalman filter.

| **Symbols** | **2011** | **2012** | **2013** | **2014** | **2015** | **2016** | **2017** | **2018** | **2019** |
| --- | --- | --- | --- | --- | --- | --- | --- | --- | --- |
| $\beta$ | 0.6917 | 0.6534 | 0.6623 | 0.7025 | 0.6731 | 0.7391 | 0.6857 | 0.7369 | 0.7507 |
| $k$ | 0.2613 | 0.2571 | 0.2509 | 0.2378 | 0.2578 | 0.2442 | 0.2639 | 0.2634 | 0.2375 |
| $\gamma_{1}$ | 3.2674 | 3.1838 | 3.2182 | 3.1584 | 3.0701 | 3.1897 | 3.3079 | 3.0144 | 2.8526 |
| $\gamma_{2}$ | 3.6997 | 3.6273 | 3.3544 | 3.4517 | 4.0750 | 3.6627 | 3.5393 | 3.9583 | 3.1628 |
| $\rho$ | 0.0204 | 0.0301 | 0.0347 | 0.0260 | 0.0317 | 0.0356 | 0.0431 | 0.0387 | 0.0645 |

**Forecasting accuracy of HFMD inpatients and outpatients**

## **Table D. Forecasting RMSE accuracy of HFMD inpatients from 2011 to 2019.** Root mean square error (RMSE) values for HFMD inpatient forecasts from 2011 to 2019, assessed **for** forecasting start months from May to December.

| **Year** | **Forecasting start month** | | | | | | | |
| --- | --- | --- | --- | --- | --- | --- | --- | --- |
|  | **5** | **6** | **7** | **8** | **9** | **10** | **11** | **12** |
| 2011 | 444.52 | 50.74 | 24.15 | 29.24 | 25.56 | 25.81 | 17.80 | 14.96 |
| 2012 | 558.43 | 266.06 | 233.88 | 178.86 | 141.02 | 98.18 | 11.90 | 10.72 |
| 2013 | 573.08 | 308.67 | 260.77 | 151.67 | 107.31 | 65.73 | 3.93 | 3.50 |
| 2014 | 730.56 | 392.77 | 453.12 | 285.39 | 125.36 | 43.77 | 27.77 | 25.65 |
| 2015 | 498.79 | 366.92 | 225.21 | 148.27 | 108.68 | 63.33 | 96.76 | 10.35 |
| 2016 | 1643.87 | 274.92 | 196.52 | 115.87 | 42.06 | 129.79 | 87.43 | 43.17 |
| 2017 | 1112.19 | 518.95 | 344.10 | 273.72 | 59.56 | 90.88 | 110.44 | 32.76 |
| 2018 | 866.33 | 290.02 | 191.91 | 197.06 | 103.38 | 135.36 | 138.24 | 49.30 |
| 2019 | 444.52 | 50.74 | 24.15 | 29.24 | 25.56 | 25.81 | 17.80 | 14.96 |

## **Table E. Forecasting RMSE accuracy of HFMD outpatients from 2011 to 2019.** RMSE values for HFMD outpatient forecasts from 2011 to 2019, evaluated for forecasting start months from May to December.

| **Year** | **Forecasting start month** | | | | | | | |
| --- | --- | --- | --- | --- | --- | --- | --- | --- |
|  | **5** | **6** | **7** | **8** | **9** | **10** | **11** | **12** |
| 2011 | 16926.29 | 5168.90 | 1510.96 | 809.63 | 1249.25 | 842.01 | 438.48 | 465.35 |
| 2012 | 10999.40 | 4345.89 | 4807.67 | 4665.66 | 3947.65 | 2388.98 | 199.14 | 191.38 |
| 2013 | 10279.40 | 2969.08 | 3998.38 | 2883.78 | 2252.39 | 1821.22 | 1153.01 | 99.82 |
| 2014 | 12938.04 | 2575.51 | 5997.74 | 2951.14 | 2363.48 | 1609.63 | 1255.12 | 911.42 |
| 2015 | 10087.61 | 8373.87 | 6435.27 | 5853.06 | 4369.75 | 2568.04 | 951.54 | 255.20 |
| 2016 | 30837.97 | 6989.81 | 2791.41 | 3927.42 | 2578.99 | 1171.50 | 653.17 | 550.34 |
| 2017 | 20349.45 | 7625.67 | 4689.04 | 3566.04 | 1738.81 | 1963.41 | 2231.23 | 624.66 |
| 2018 | 21880.16 | 6197.68 | 5503.84 | 3643.80 | 2945.34 | 1564.45 | 1289.77 | 1256.67 |
| 2019 | 53396.18 | 9103.37 | 3731.49 | 4283.16 | 2216.14 | 1504.07 | 1284.77 | 974.11 |

## **Table F. Forecasting correlation accuracy of HFMD inpatients from 2011 to 2019.** Correlation coefficients between observed and forecasted HFMD inpatient data from 2011 to 2019, calculated for forecasting start months from May to December.

| **Year** | **Forecasting start month** | | | | | | | |
| --- | --- | --- | --- | --- | --- | --- | --- | --- |
|  | **5** | **6** | **7** | **8** | **9** | **10** | **11** | **12** |
| 2011 | 0.8927 | 0.9991 | 0.9998 | 0.9996 | 0.9997 | 0.9997 | 0.9999 | 1.0000 |
| 2012 | 0.7007 | 0.9534 | 0.9626 | 0.9731 | 0.9815 | 0.9890 | 0.9999 | 1.0000 |
| 2013 | 0.8358 | 0.9586 | 0.9733 | 0.9893 | 0.9942 | 0.9969 | 1.0000 | 1.0000 |
| 2014 | 0.8262 | 0.9557 | 0.9386 | 0.9757 | 0.9954 | 0.9992 | 0.9999 | 0.9999 |
| 2015 | 0.5871 | 0.7900 | 0.9278 | 0.9698 | 0.9756 | 0.9901 | 0.9781 | 0.9999 |
| 2016 | 0.8134 | 0.9949 | 0.9974 | 0.9990 | 0.9999 | 0.9990 | 0.9996 | 0.9999 |
| 2017 | 0.4496 | 0.9097 | 0.9571 | 0.9748 | 0.9991 | 0.9972 | 0.9958 | 0.9999 |
| 2018 | 0.5002 | 0.9628 | 0.9821 | 0.9776 | 0.9932 | 0.9890 | 0.9894 | 0.9999 |
| 2019 | 0.6094 | 0.9902 | 0.9959 | 0.9986 | 0.9996 | 0.9993 | 0.9996 | 0.9999 |

## **Table G. Forecasting correlation accuracy of HFMD outpatients from 2011 to 2019.** Correlation coefficients between observed and forecasted HFMD outpatient data from 2011 to 2019, computed for forecasting start months from May to December.

| **Year** | **Forecasting start month** | | | | | | | |
| --- | --- | --- | --- | --- | --- | --- | --- | --- |
|  | **5** | **6** | **7** | **8** | **9** | **10** | **11** | **12** |
| 2011 | 0.9175 | 0.9946 | 0.9995 | 0.9998 | 0.9996 | 0.9998 | 1.0000 | 1.0000 |
| 2012 | 0.8429 | 0.9824 | 0.9778 | 0.9735 | 0.9800 | 0.9914 | 1.0000 | 1.0000 |
| 2013 | 0.9201 | 0.9953 | 0.9916 | 0.9945 | 0.9956 | 0.9969 | 0.9989 | 1.0000 |
| 2014 | 0.9408 | 0.9977 | 0.9907 | 0.9974 | 0.9979 | 0.9990 | 0.9997 | 0.9999 |
| 2015 | 0.7832 | 0.8396 | 0.9076 | 0.9279 | 0.9498 | 0.9794 | 0.9969 | 0.9999 |
| 2016 | 0.8545 | 0.9963 | 0.9989 | 0.9980 | 0.9991 | 0.9998 | 1.0000 | 1.0000 |
| 2017 | 0.5030 | 0.9534 | 0.9774 | 0.9901 | 0.9971 | 0.9964 | 0.9955 | 1.0000 |
| 2018 | 0.5057 | 0.9733 | 0.9807 | 0.9887 | 0.9939 | 0.9980 | 0.9999 | 0.9999 |
| 2019 | 0.6223 | 0.9944 | 0.9984 | 0.9984 | 0.9994 | 0.9998 | 0.9999 | 1.0000 |

**Estimation of transmission rate from outpatients** $\mathbf{(}\boldsymbol{\beta}_{\mathbf{1}}\mathbf{)}$


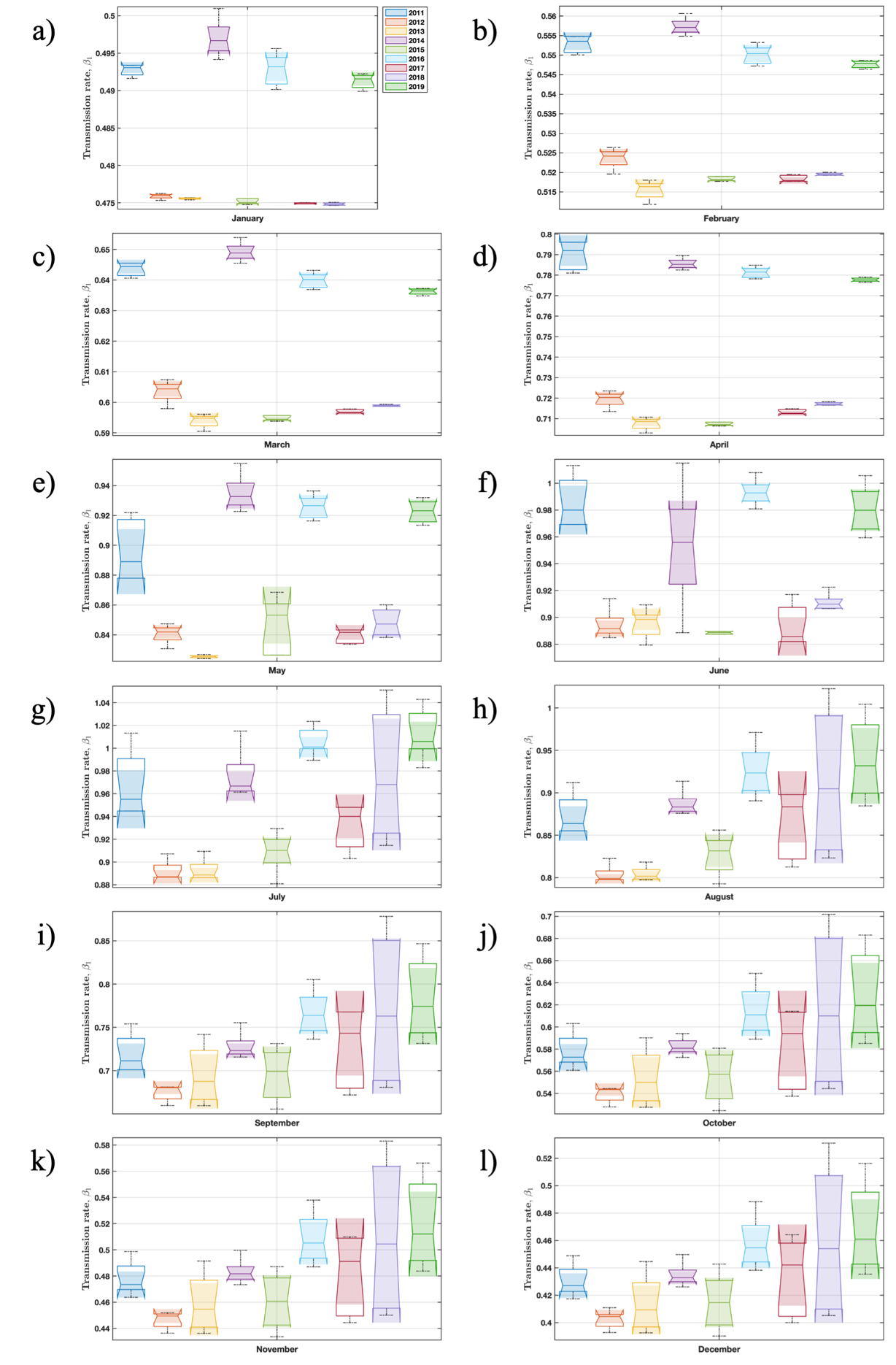


## **Fig A.** **Change in parameter** $\boldsymbol{\beta}_{\boldsymbol{1}}$**​ over the years 2011 to 2019.** Monthly variations in the transmission rate $\beta_{1}$​ (from outpatient data) for HFMD in Korea from 2011 to 2019.

**Estimation of transmission rate from inpatients** $\mathbf{(}\boldsymbol{\beta}_{\mathbf{2}}\mathbf{)}$


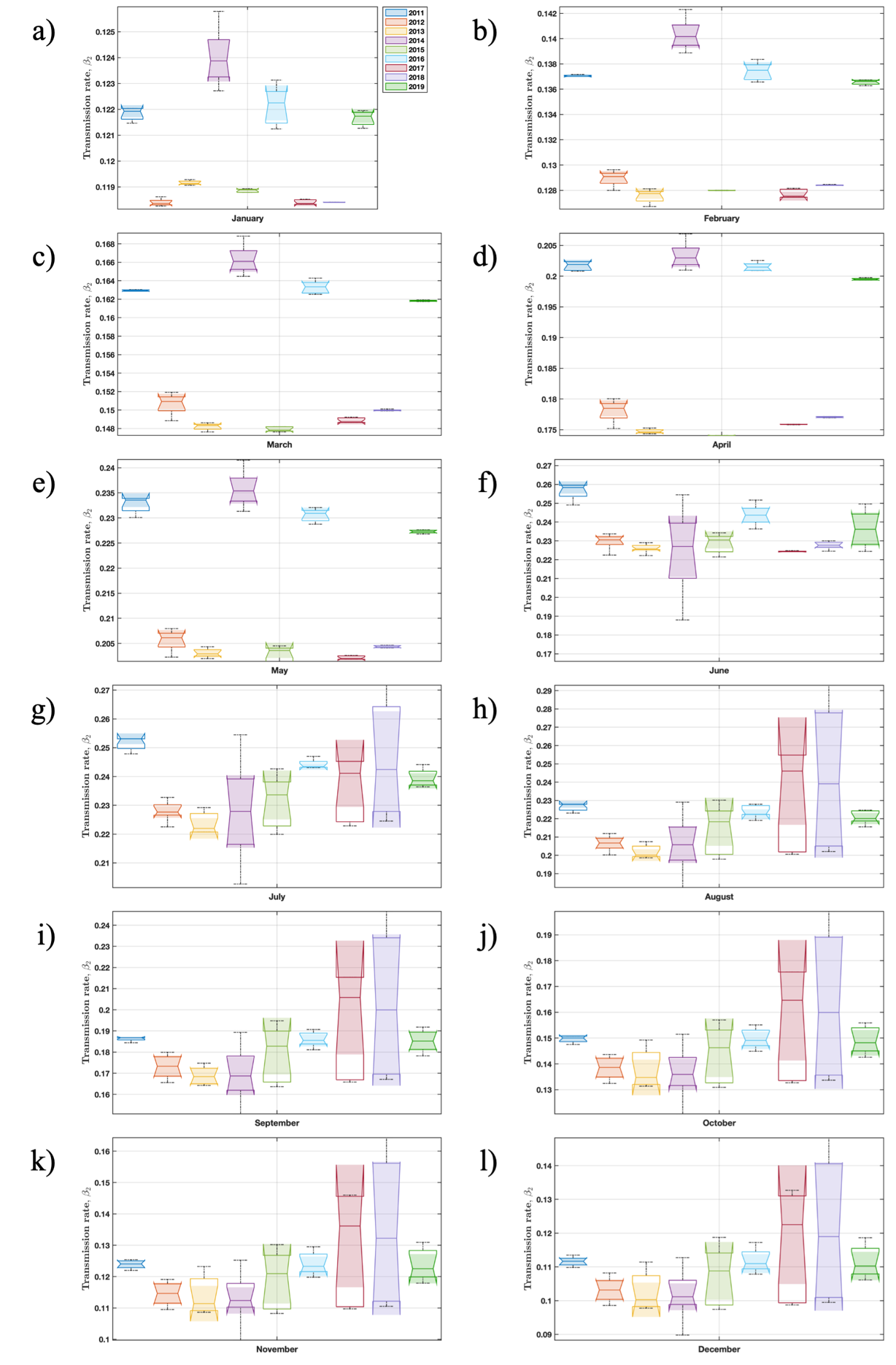


## **Fig B. Change in transmission rate** $\boldsymbol{\beta}_{\boldsymbol{2}}$ **observed from 2011 to 2019.** Monthly changes in the transmission rate $\beta_{2}$​ (from inpatient data) for HFMD in Korea from 2011 to 2019.

**Estimation of recovery rate of outpatients** $\mathbf{(}\boldsymbol{\gamma}_{\mathbf{1}}\mathbf{)}$


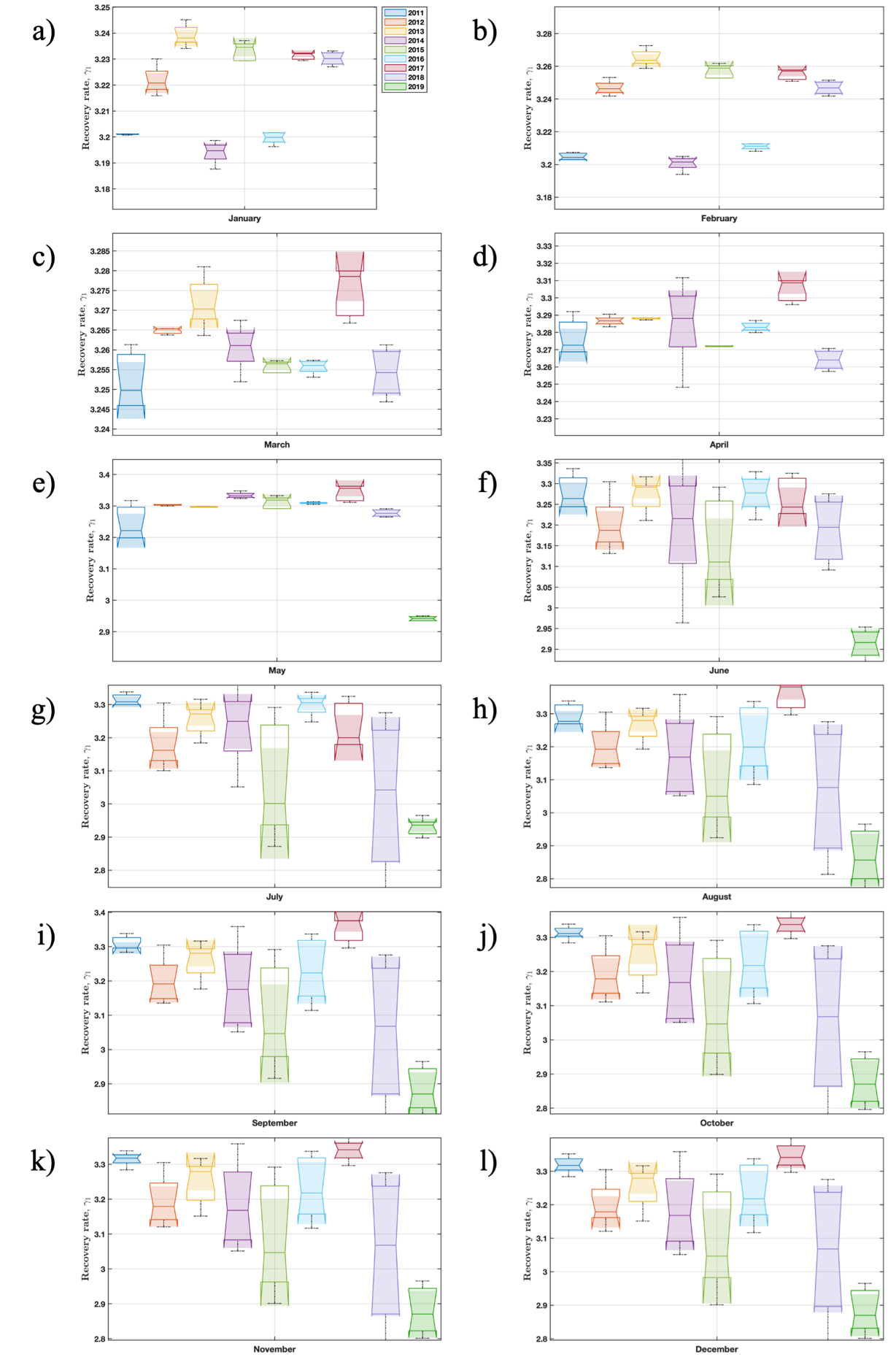


## **Fig C. Change in recovery rate** $\boldsymbol{\gamma}_{\boldsymbol{1}}$ **observed from 2011 to 2019.** Monthly variations in the recovery rate $\gamma_{1}$​ (for outpatients) for HFMD in Korea from 2011 to 2019.

**Estimation of recovery rate of inpatients** $\mathbf{(}\boldsymbol{\gamma}_{\mathbf{2}}\mathbf{)}$


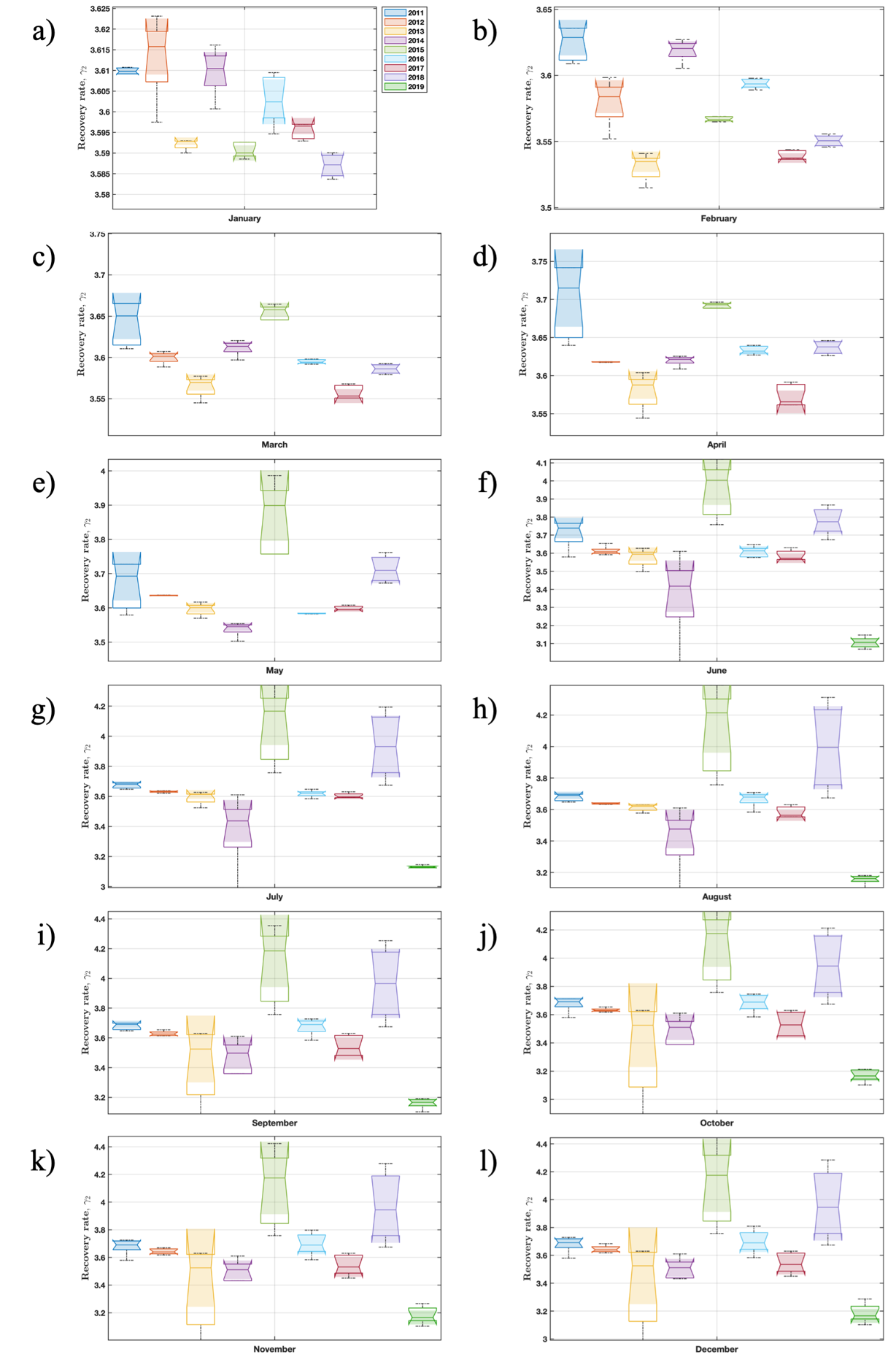


## **Fig D. Change in recovery rate** $\boldsymbol{\gamma}_{\boldsymbol{2}}$ **observed from 2011 to 2019.** Monthly variations in the recovery rate $\gamma_{2}$​ (for inpatients) for HFMD in Korea from 2011 to 2019.

**Real-time forecasting and fitting results of HFMD inpatients and outpatients**

The analysis in this study focuses on examining the trends in HFMD (Hand, Foot, and Mouth Disease) cases among inpatients and outpatients in the years 2012 and 2018. Monthly observations of both inpatients and outpatients are represented by data points. The forecasting is done based on the data from previous months. The data points for outpatients are represented by green circles, while inpatient data is denoted by brown circles. Forecasts derived from these datasets are indicated by red circles. The dashed lines illustrate the fit and forecast for each year, providing valuable insights into the progression of the disease and the accuracy of the forecasting model over different periods from 2012 to 2018.


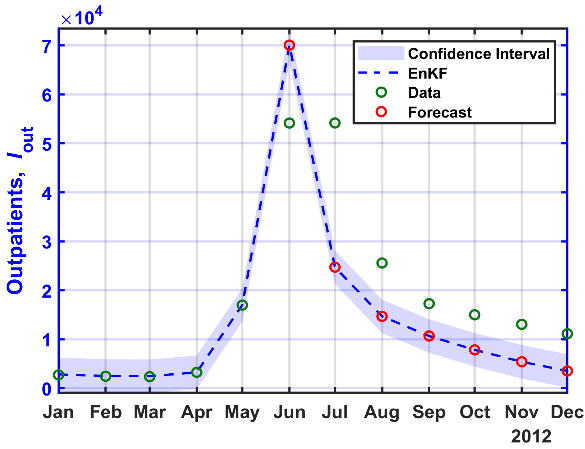

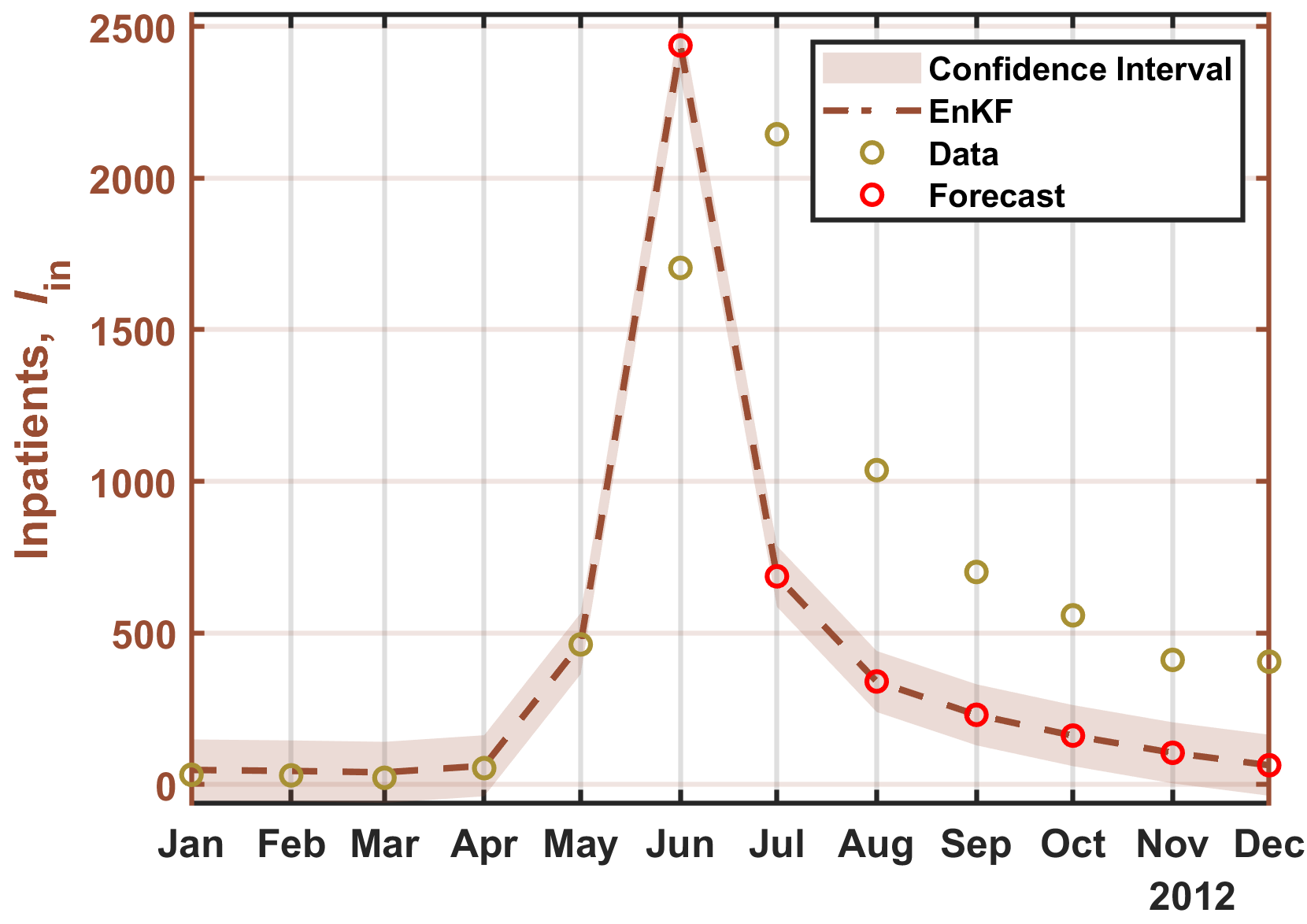


**a)**


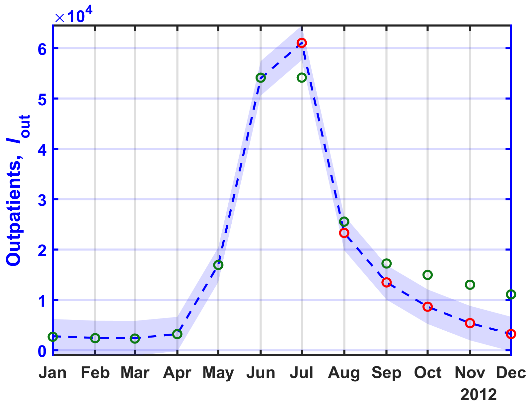

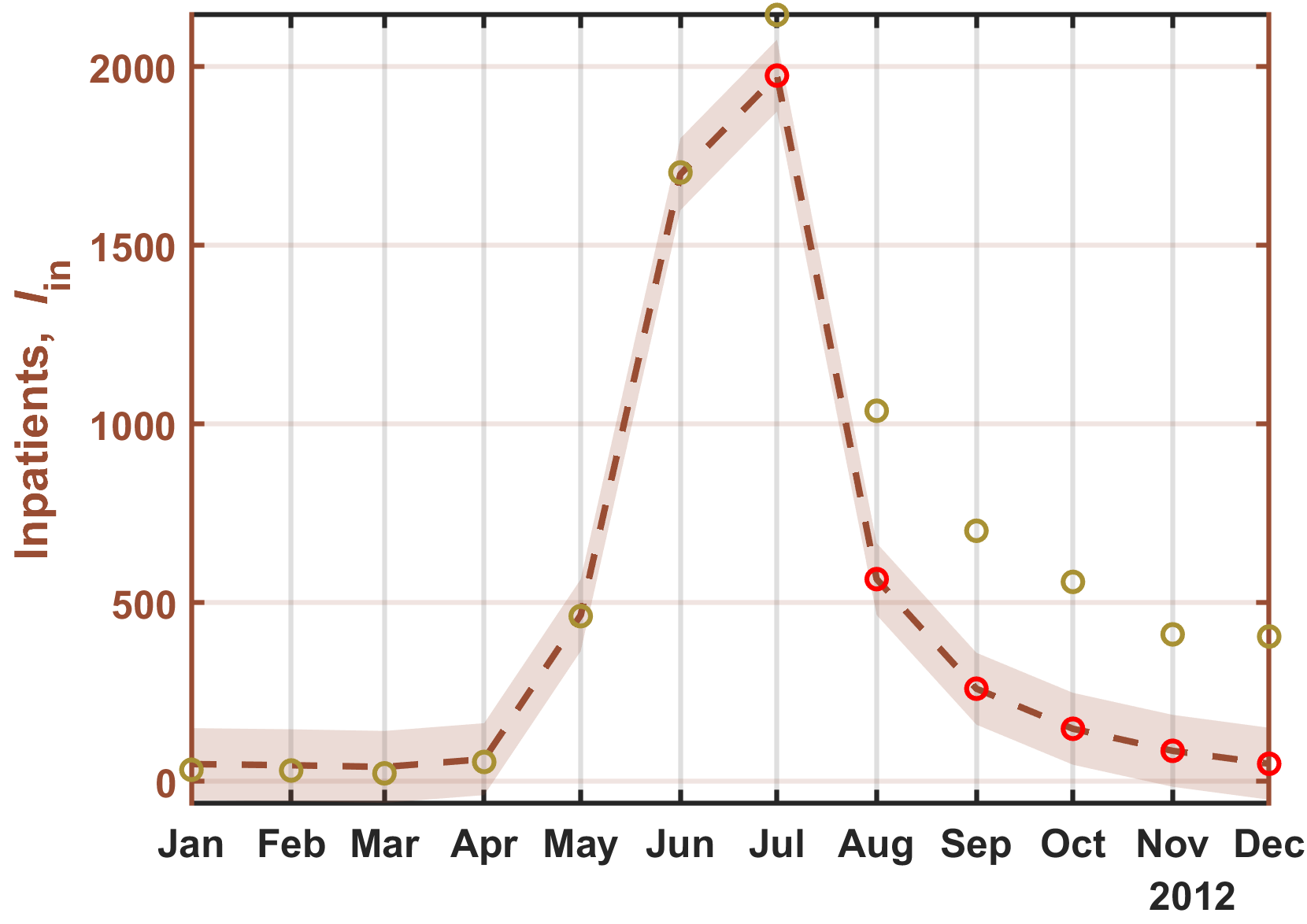


**b)**


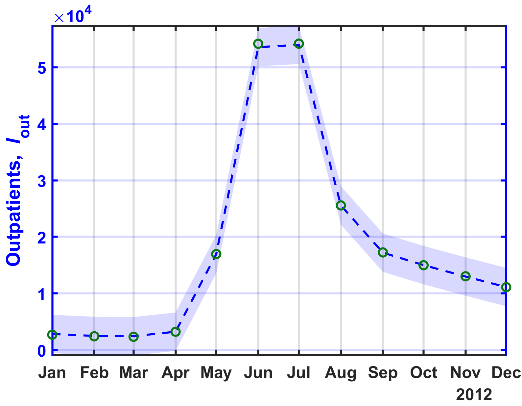

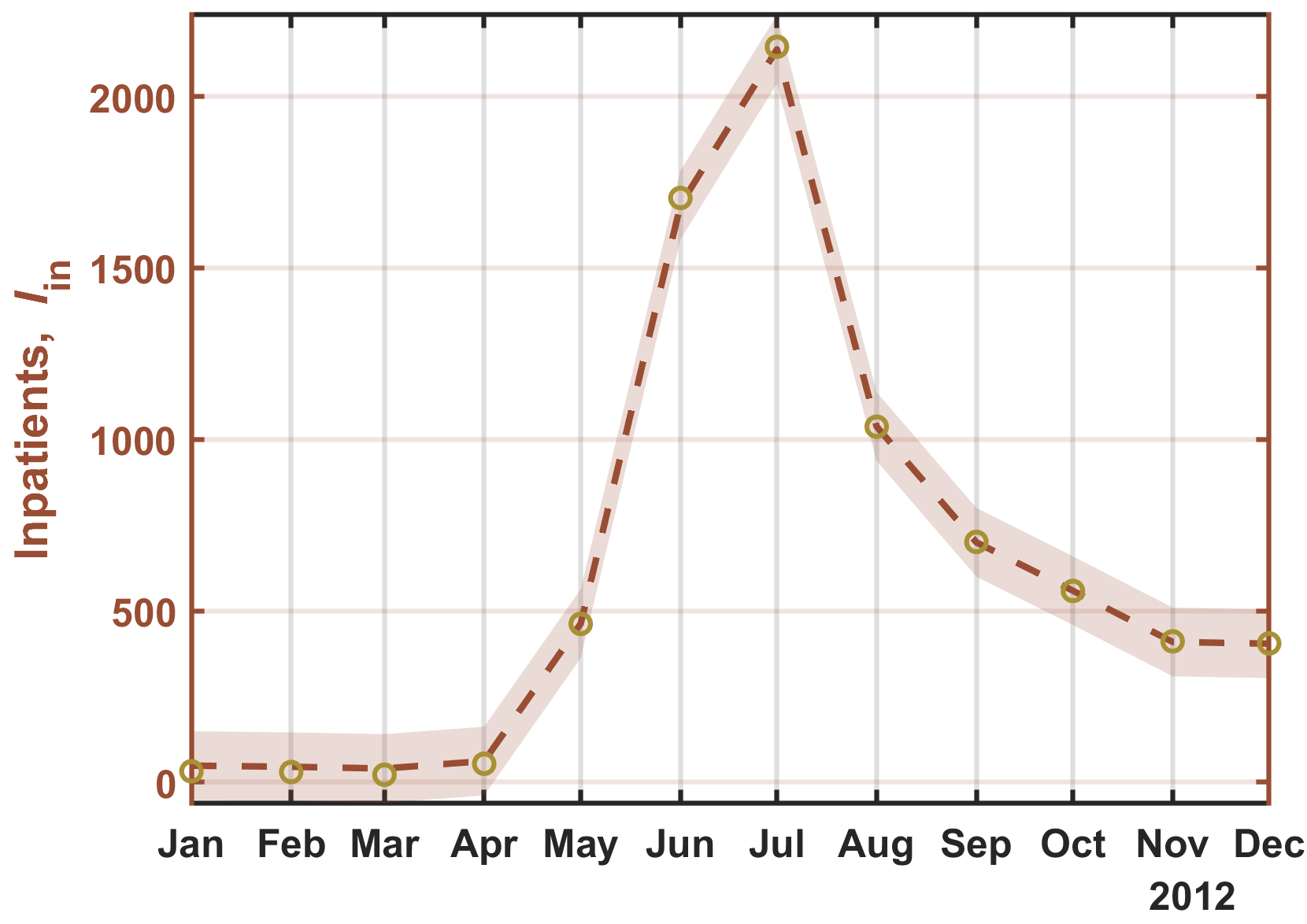


**c)**

## **Fig E. Real-time estimation and forecasting results of HFMD inpatients and outpatients in 2012.** Real-time estimation and forecasting of HFMD inpatients (brown circles) and outpatients (green circles) in 2012, with forecasts (red circles) and fitted trends (dashed lines).


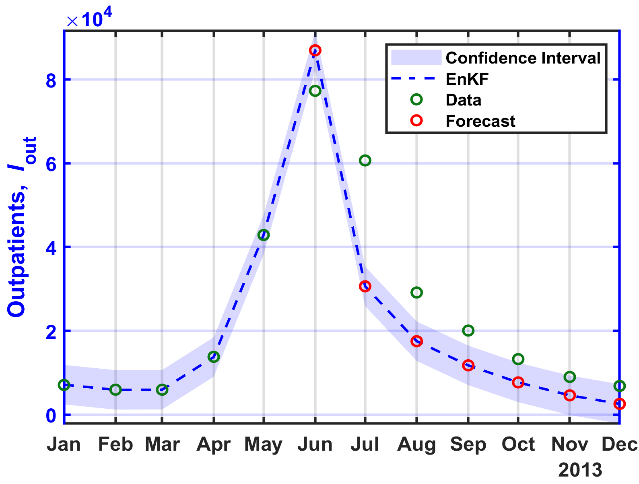

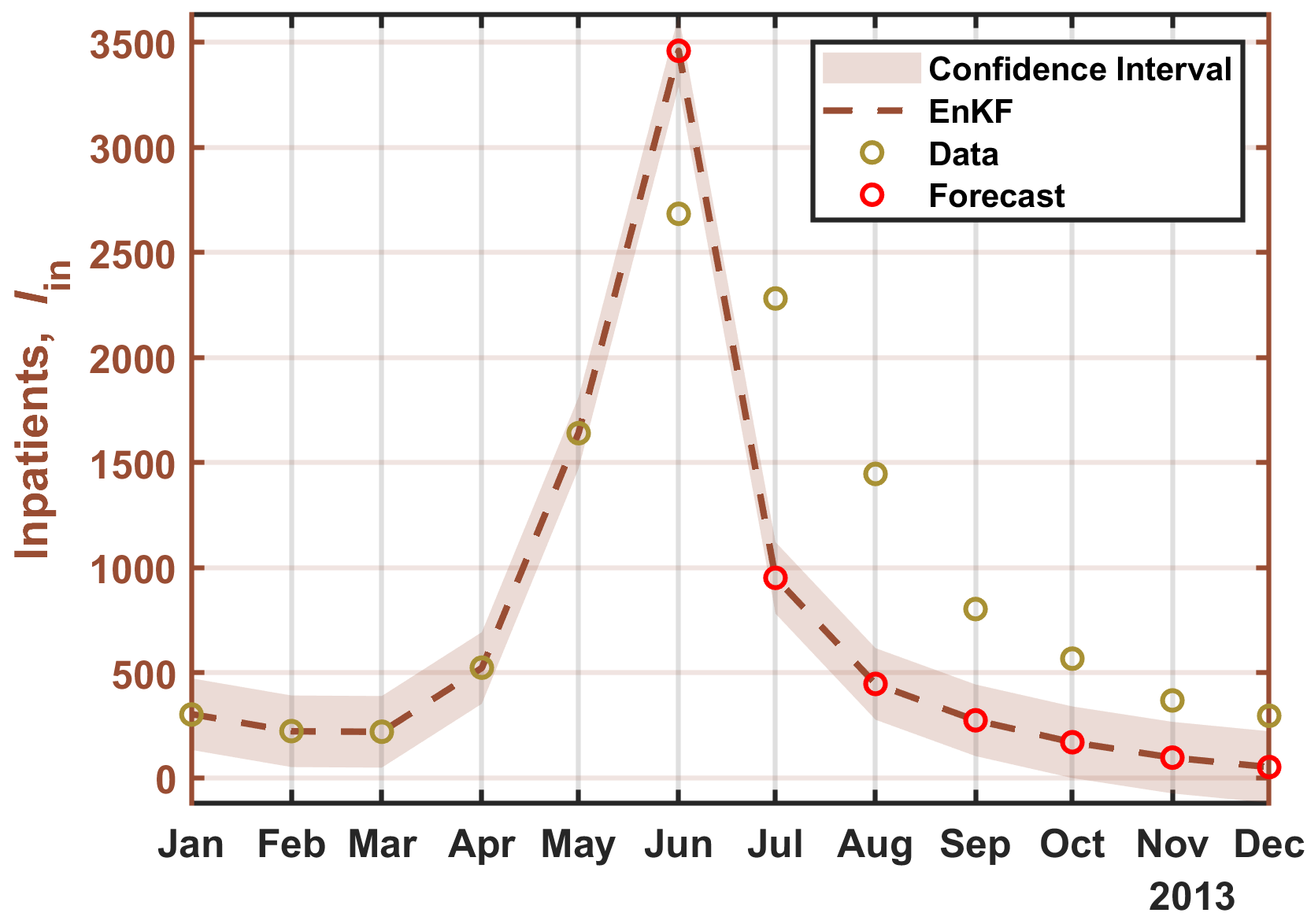


**a)**


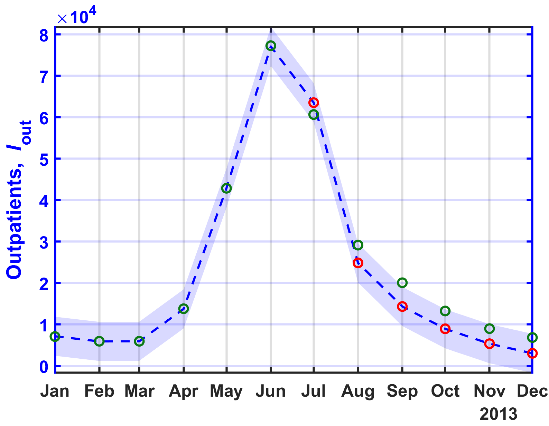

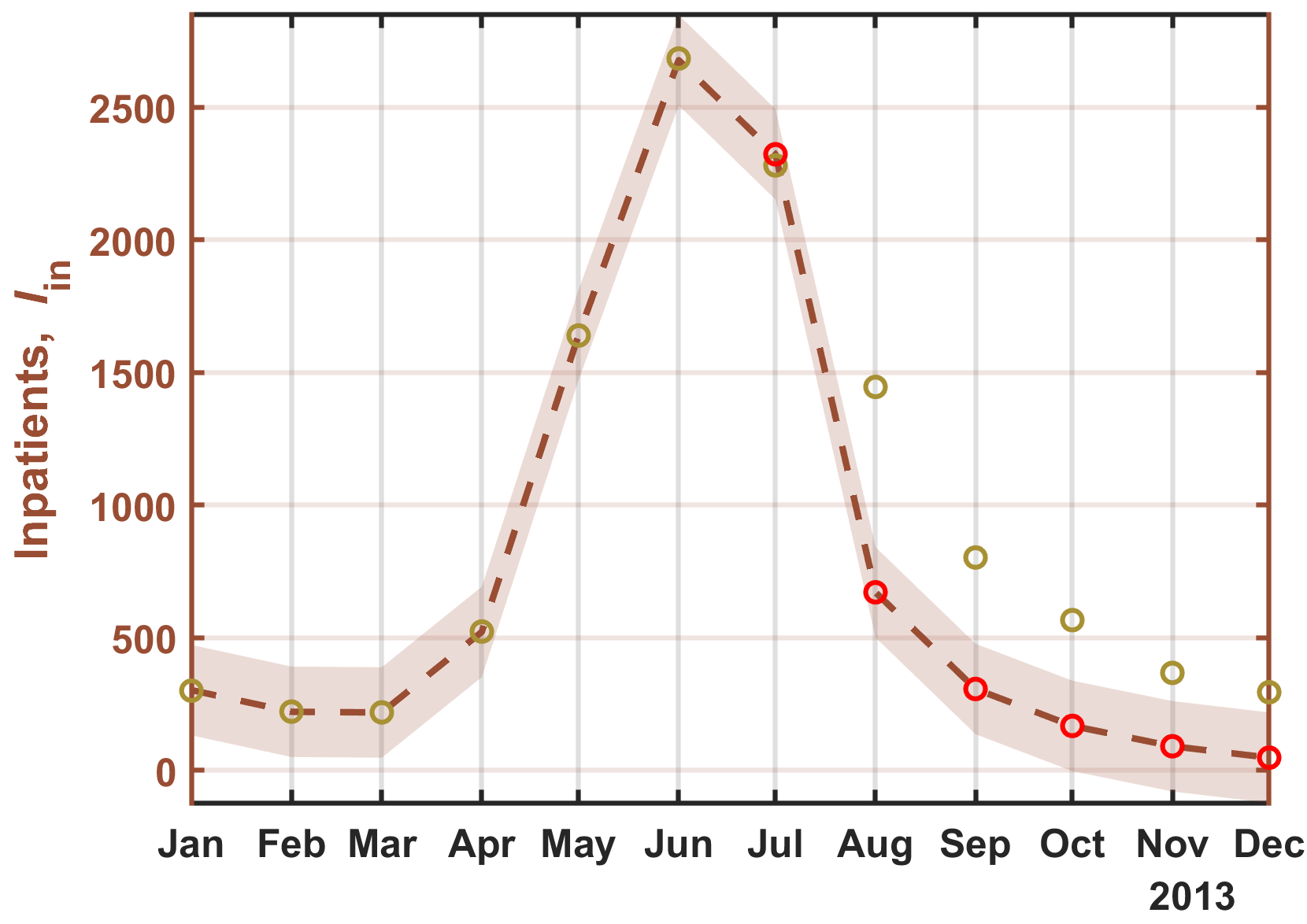


**b)**


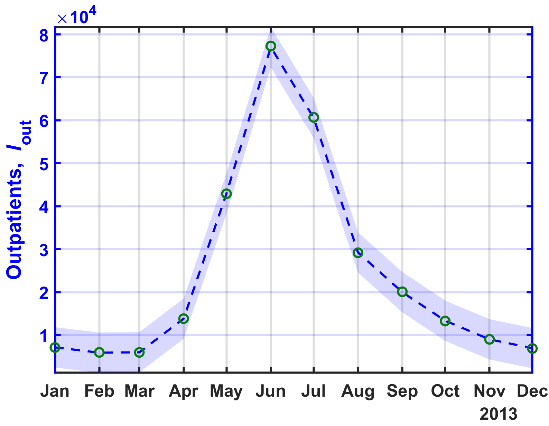

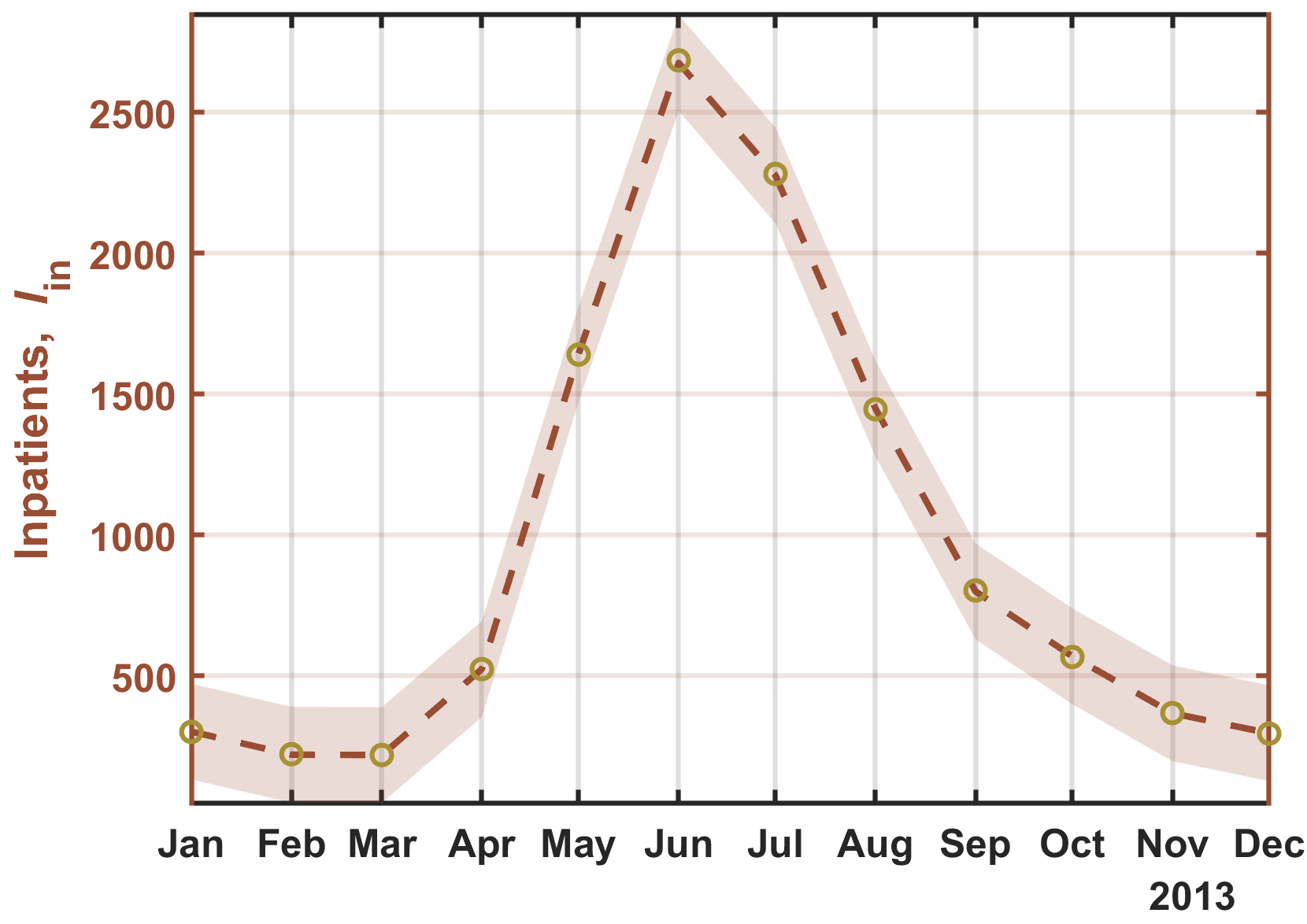


**c)**

## **Fig F. Real-time estimation and forecasting results of HFMD inpatients and outpatients in 2013.** Real-time estimation and forecasting of HFMD inpatients and outpatients in 2013, following the format of Fig E.


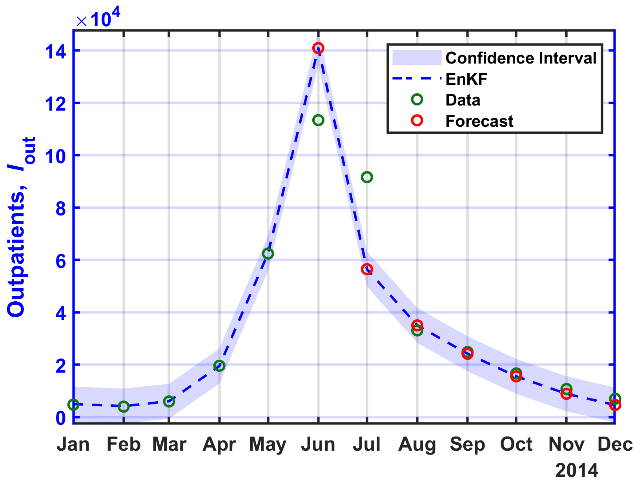

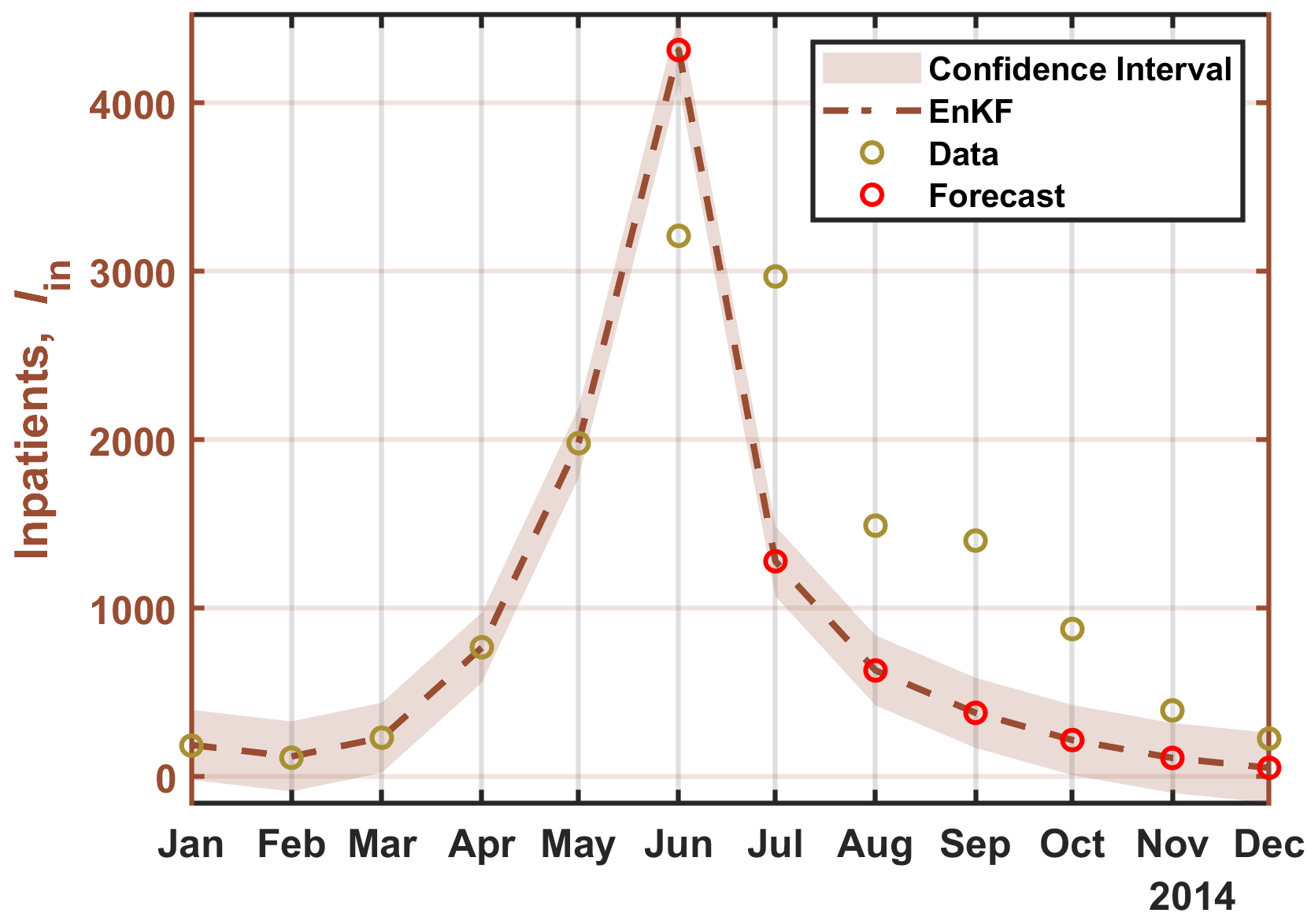


**a)**


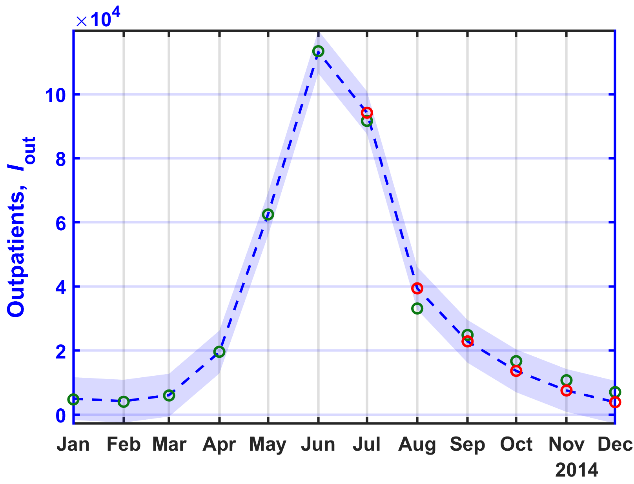

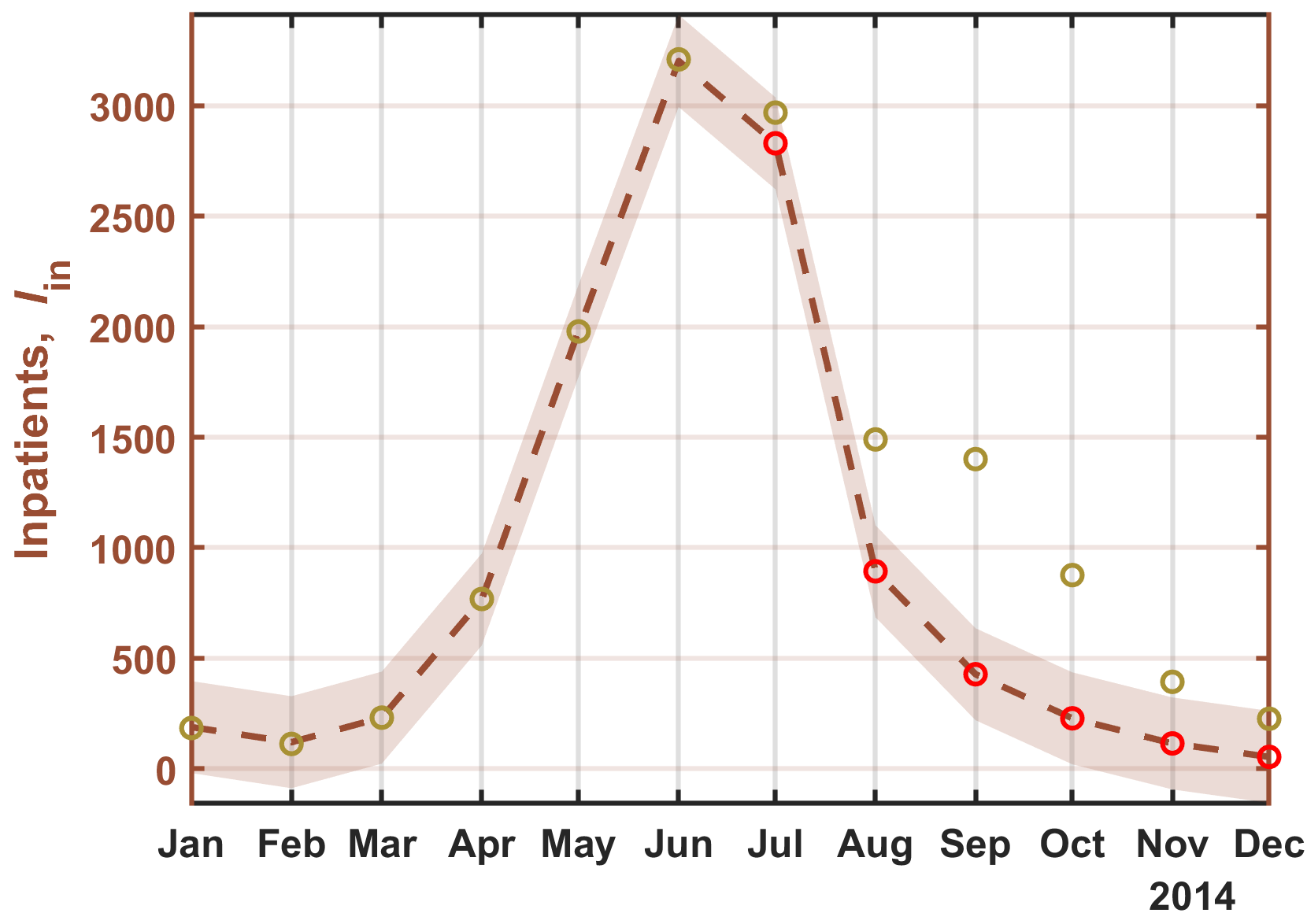


**b)**


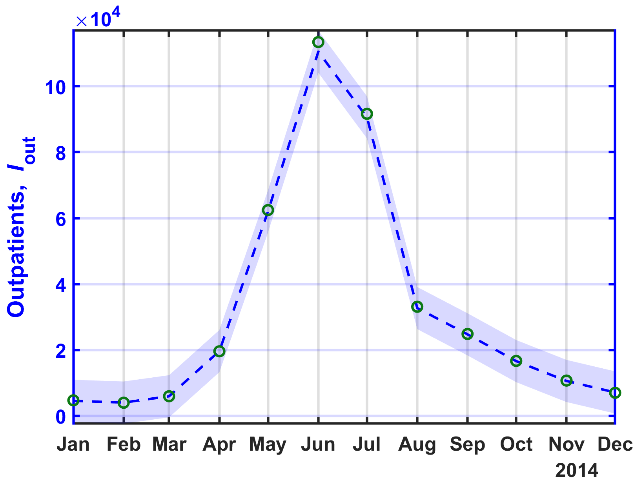

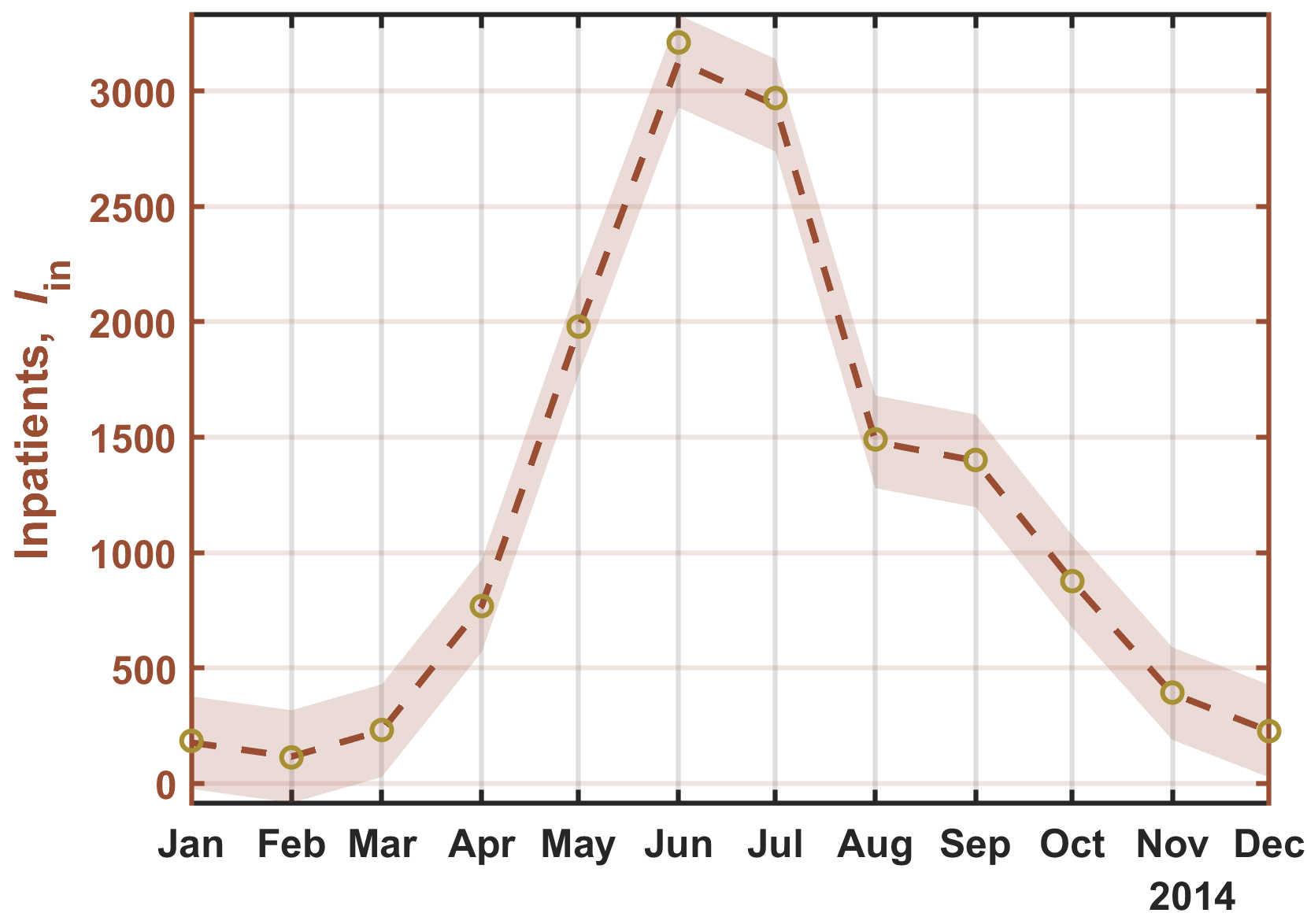


**c)**

## **Fig G. Real-time estimation and forecasting results of HFMD inpatients and outpatients in 2014.** Real-time estimation and forecasting of HFMD inpatients and outpatients in 2014, consistent with Fig E.

## **Fig H. Real-time estimation and forecasting results of HFMD inpatients and outpatients in 2015.** Real-time estimation and forecasting of HFMD inpatients and outpatients in 2015, adhering to the style of Fig E.


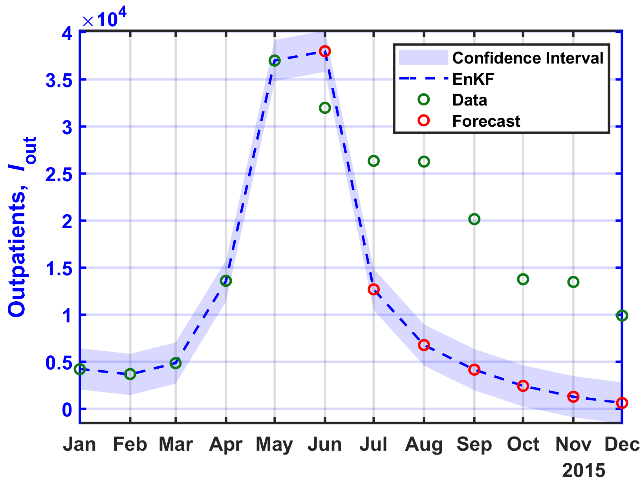

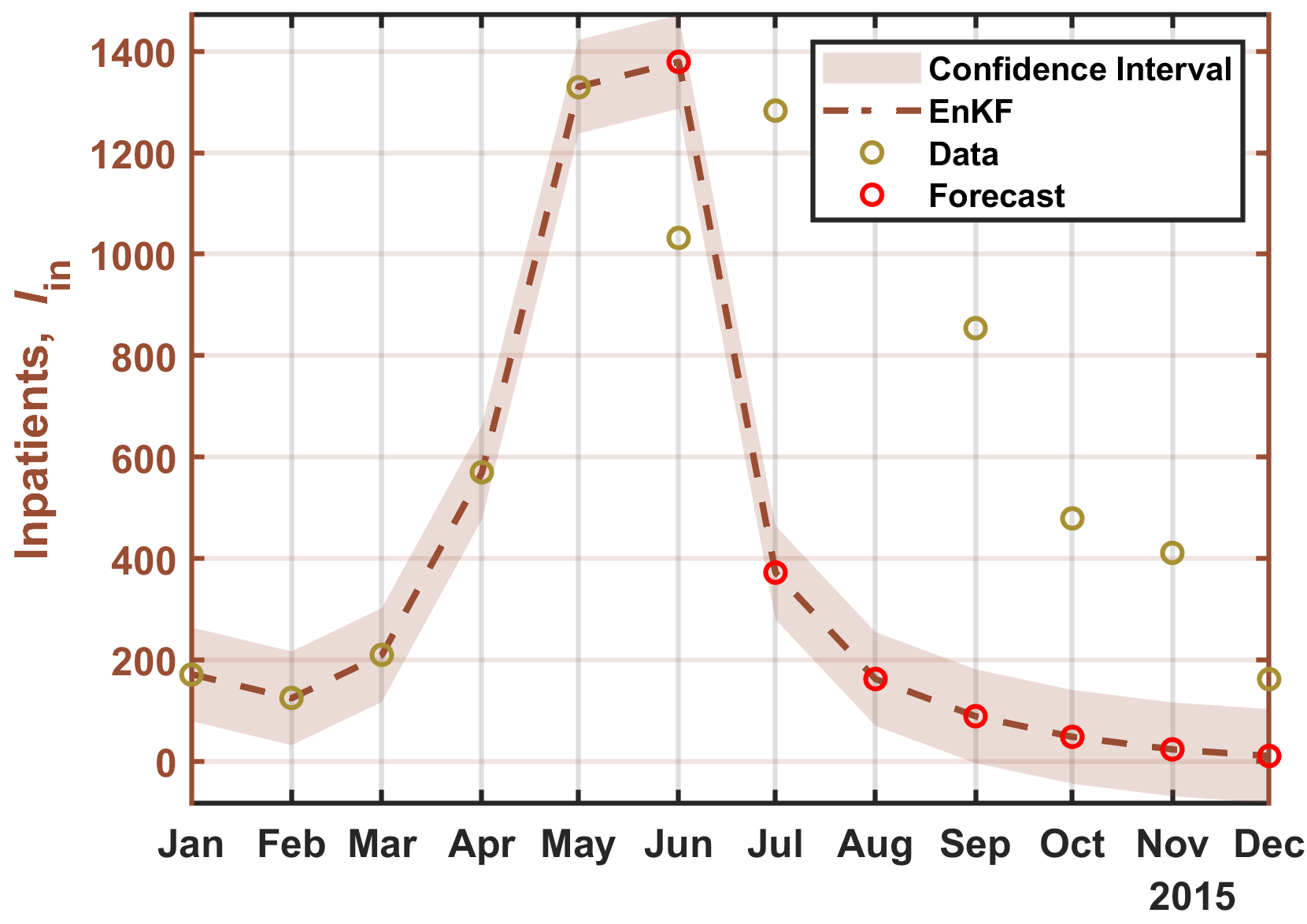


**a)**


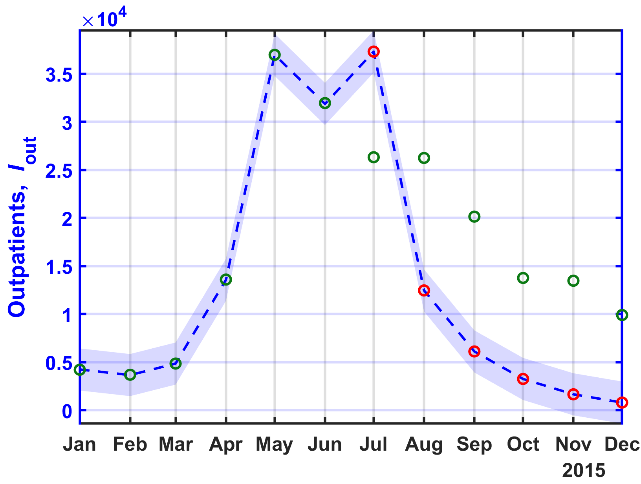

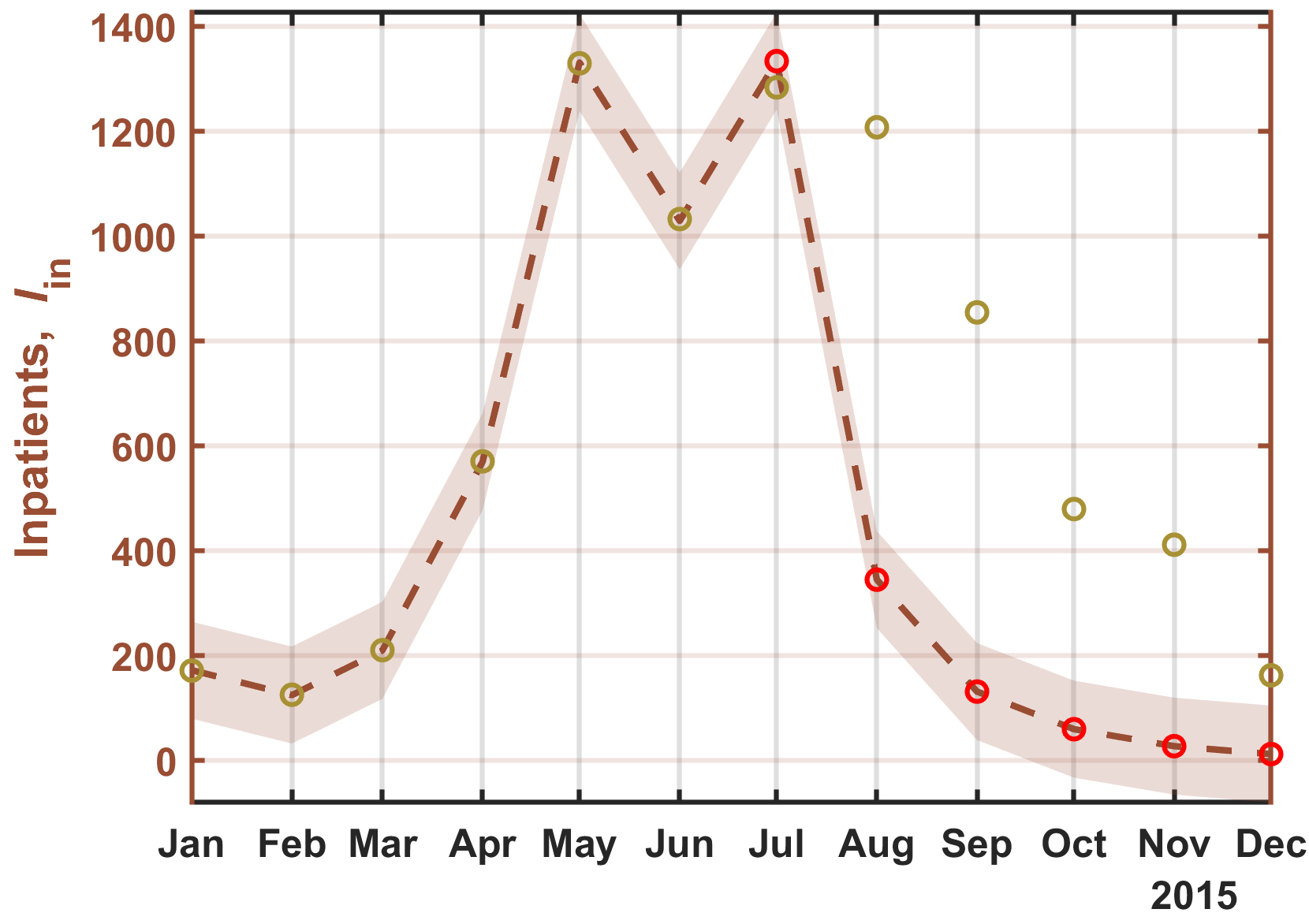


**b)**


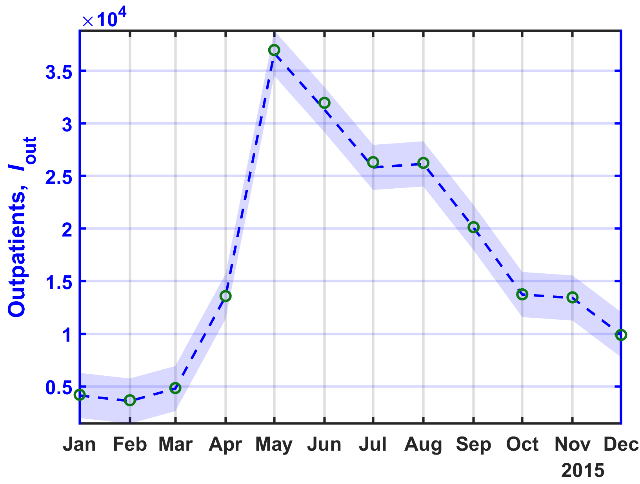

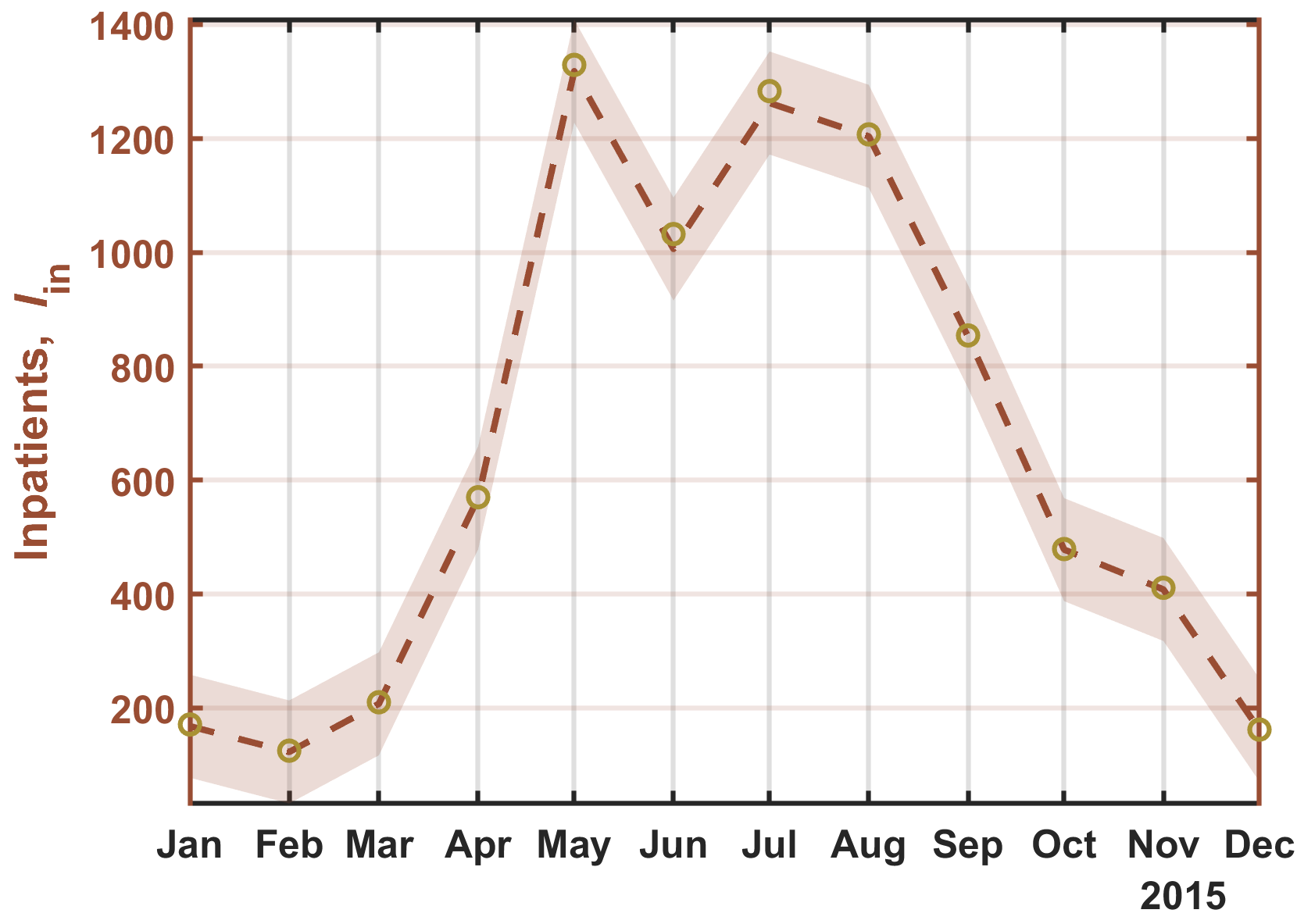


**c)**


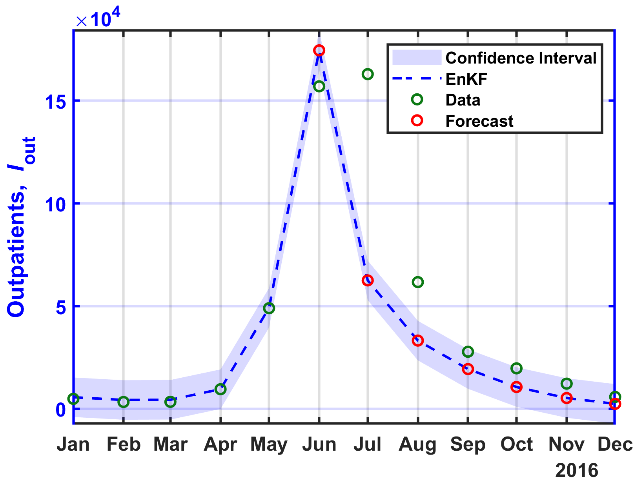

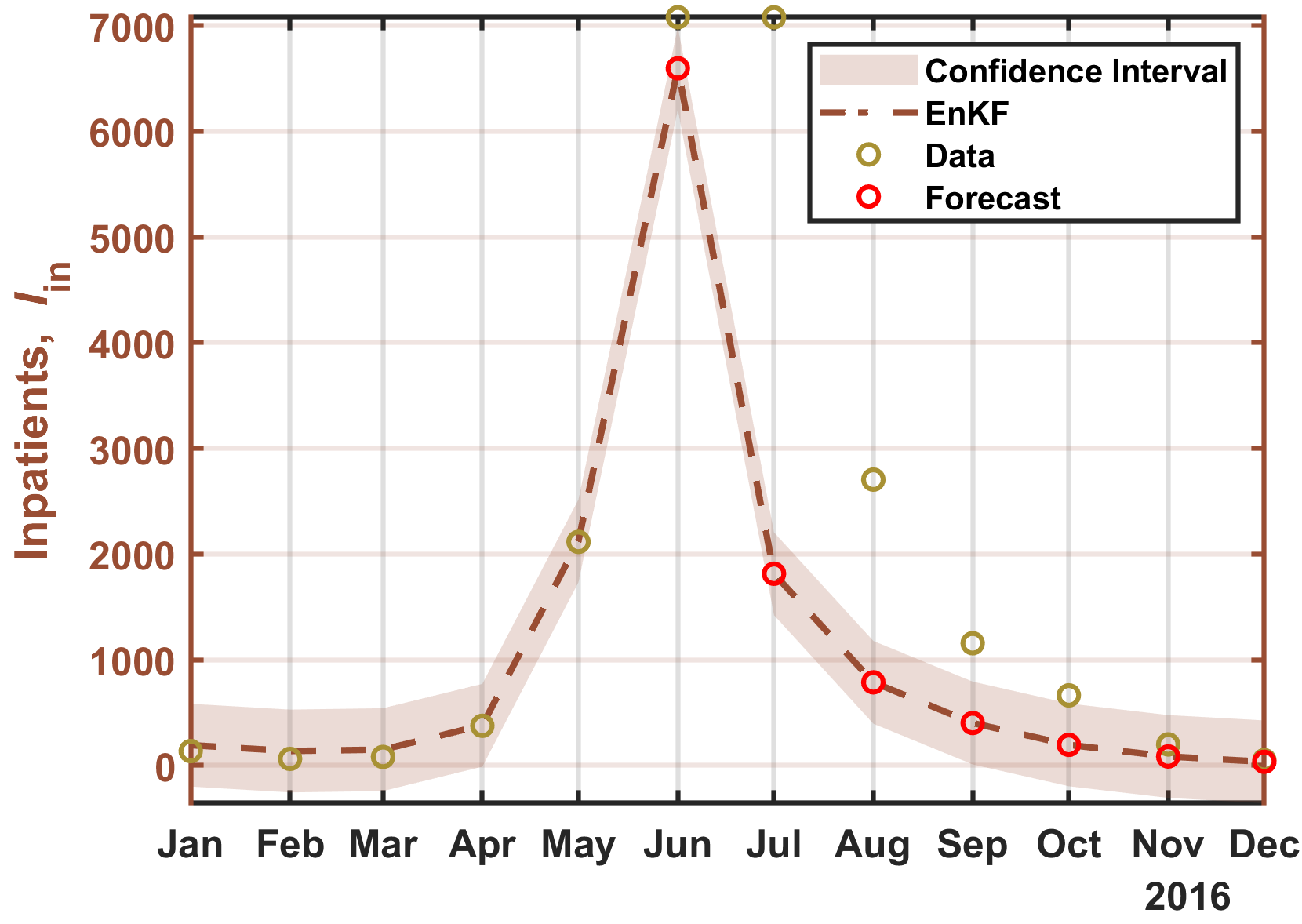


**a)**


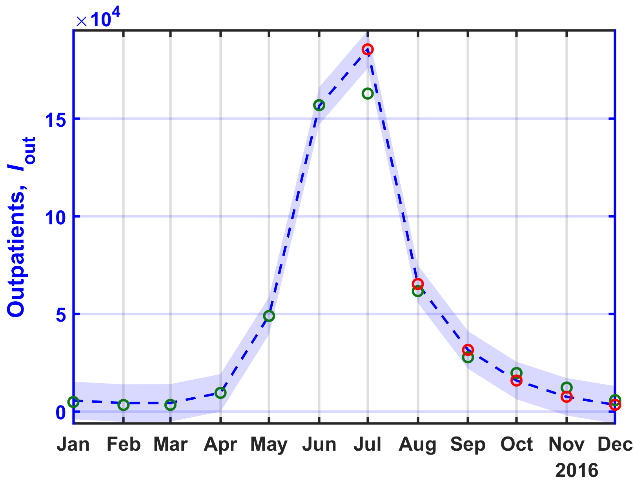

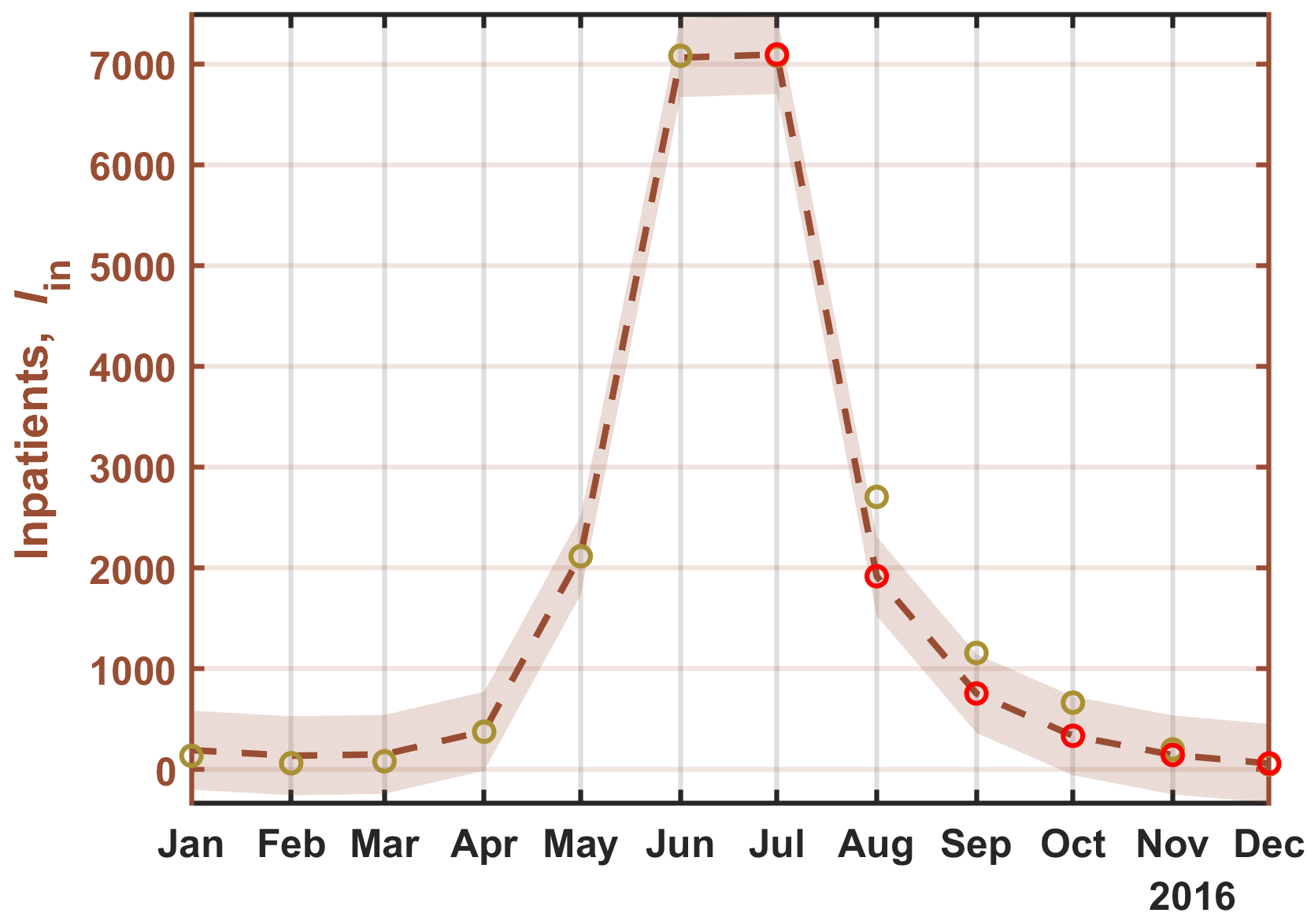


**b)**


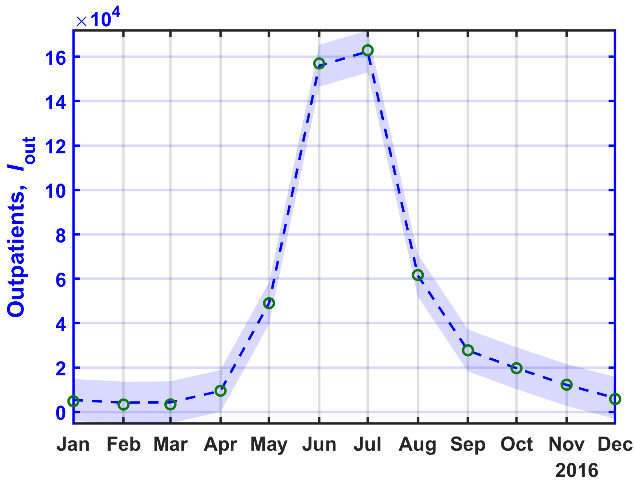

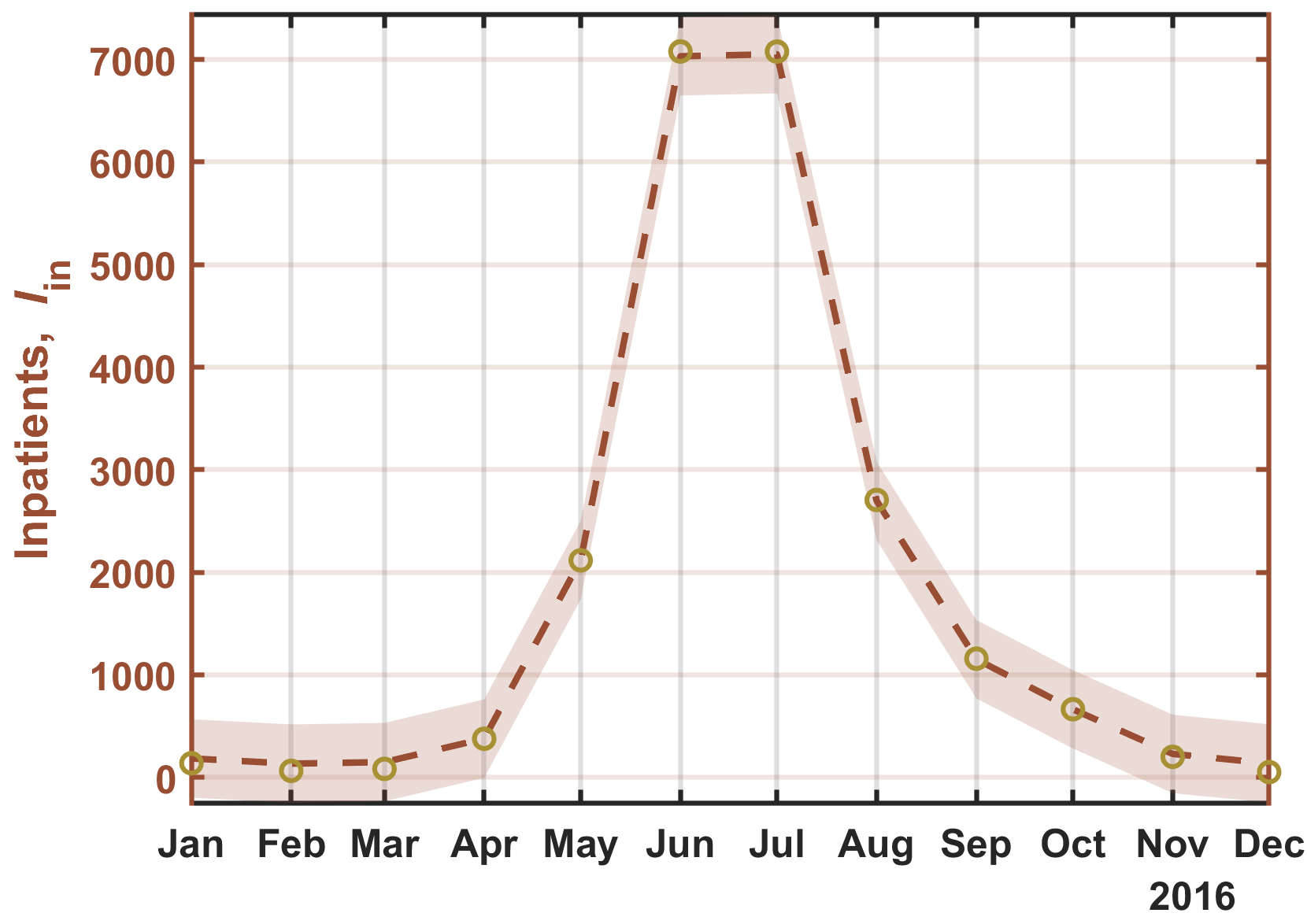


**c)**

## **Fig I. Real-time estimation and forecasting results of HFMD inpatients and outpatients in 2016.** Real-time estimation and forecasting of HFMD inpatients and outpatients in 2016, matching the format of Fig E.


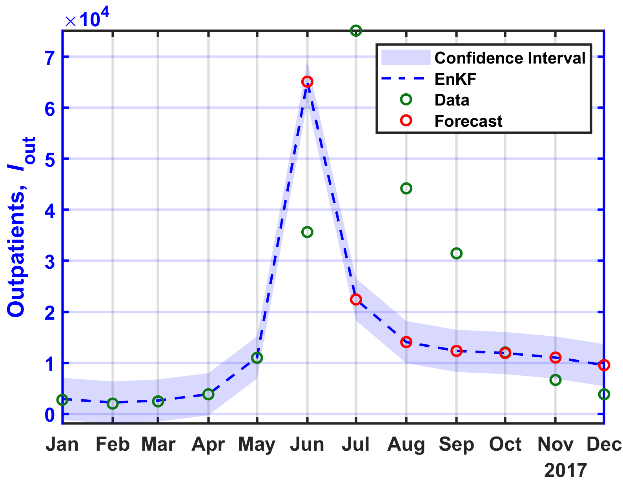

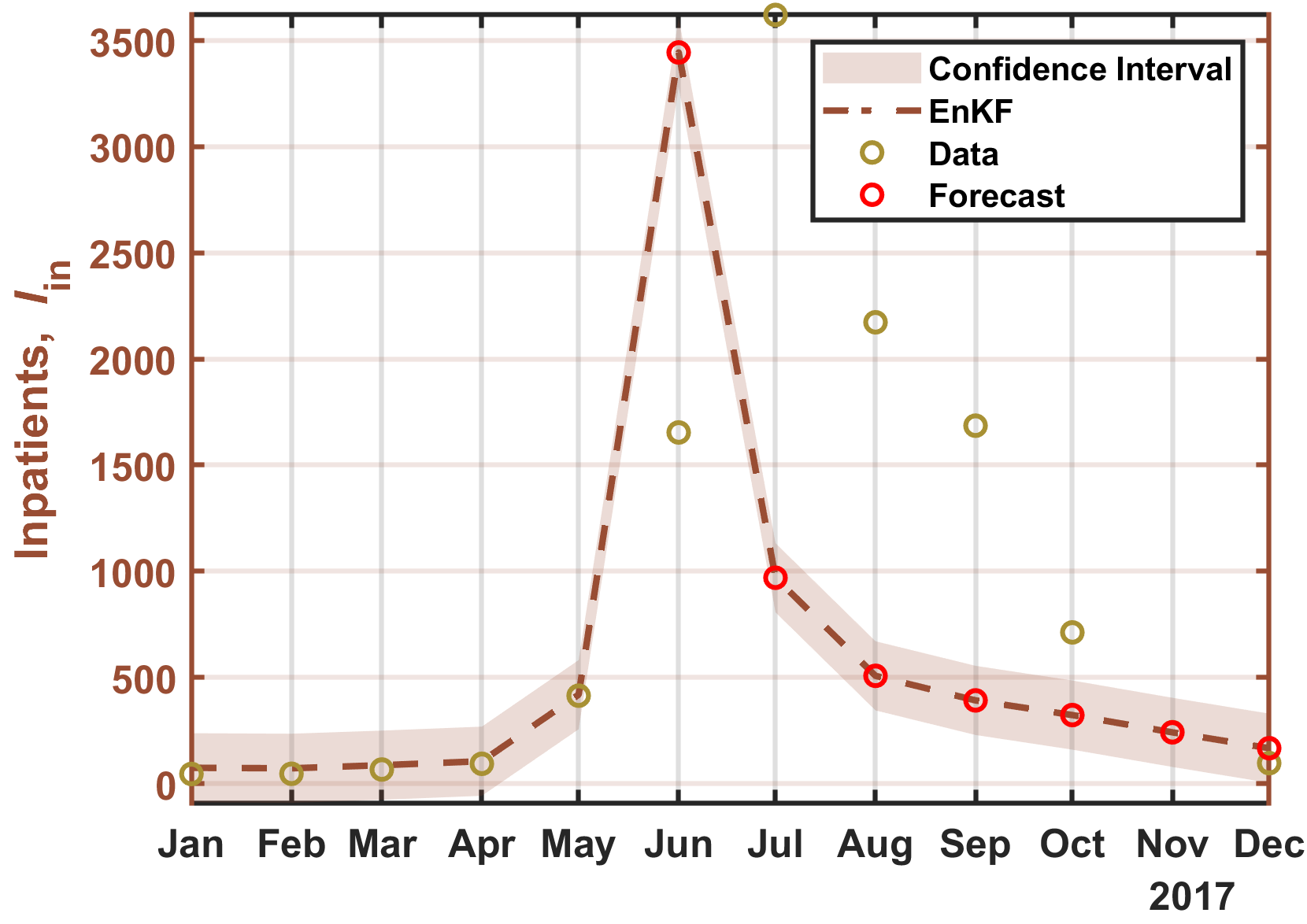


**a)**


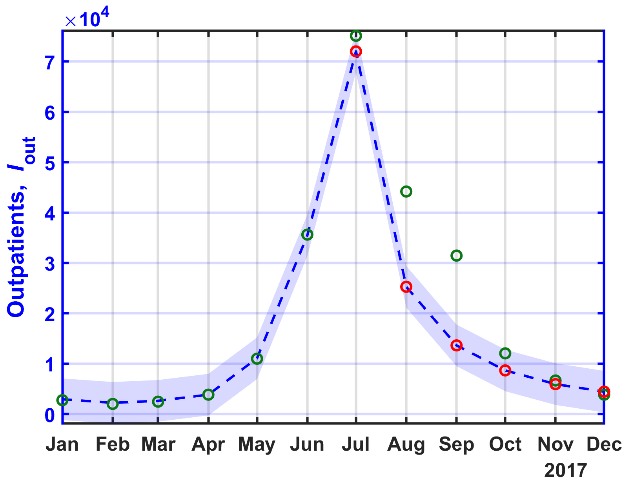

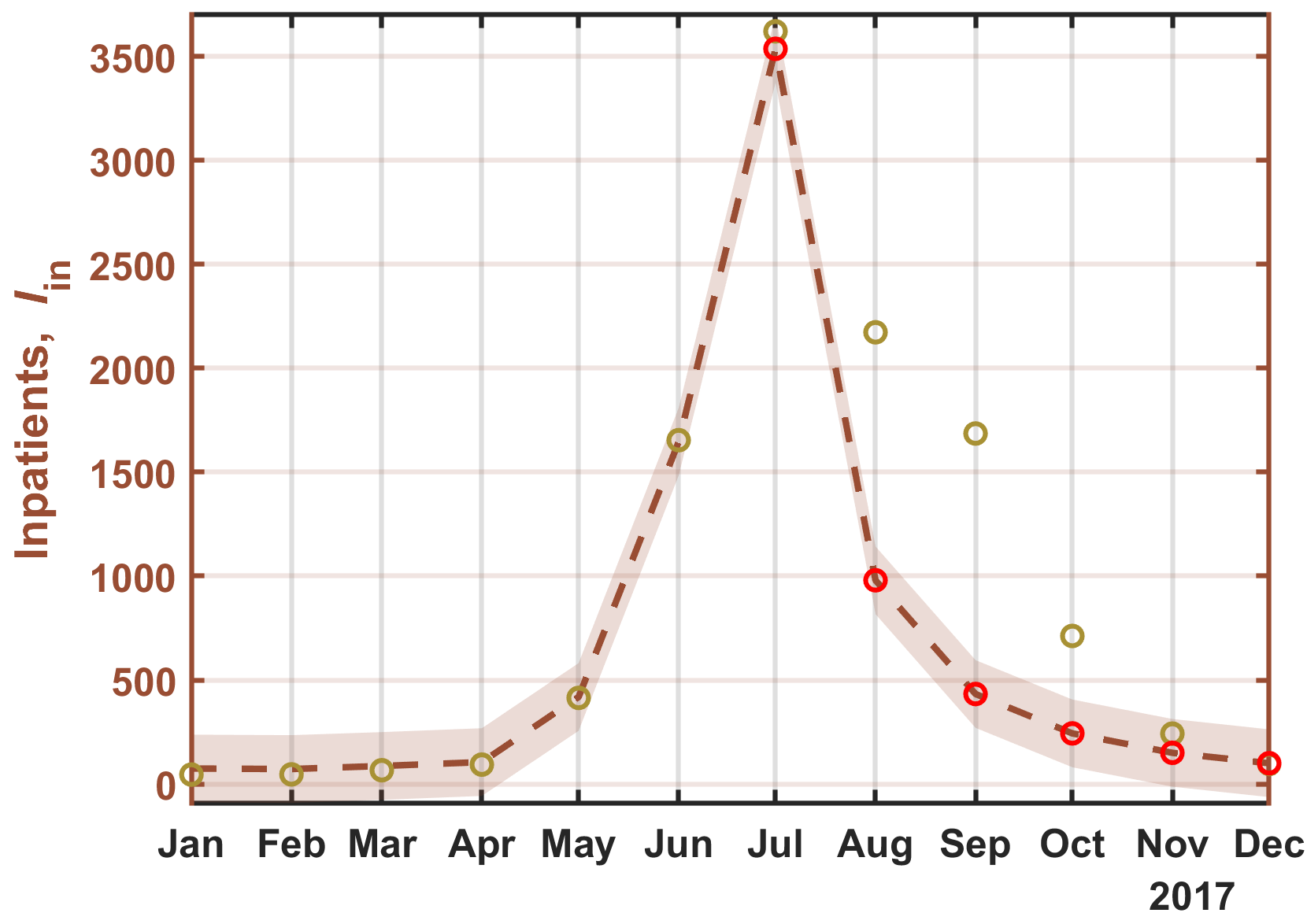


**b)**


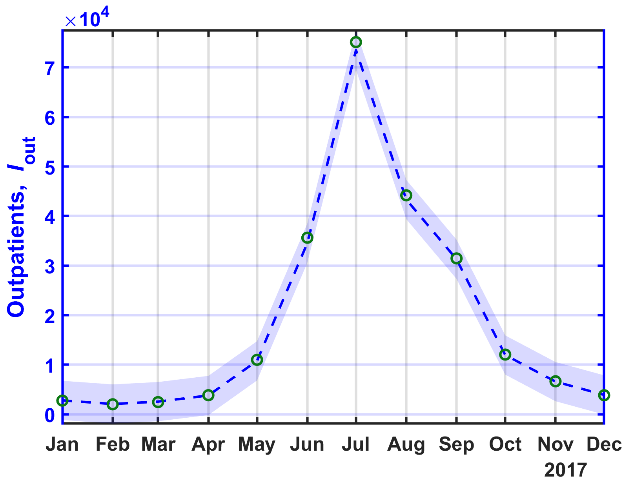

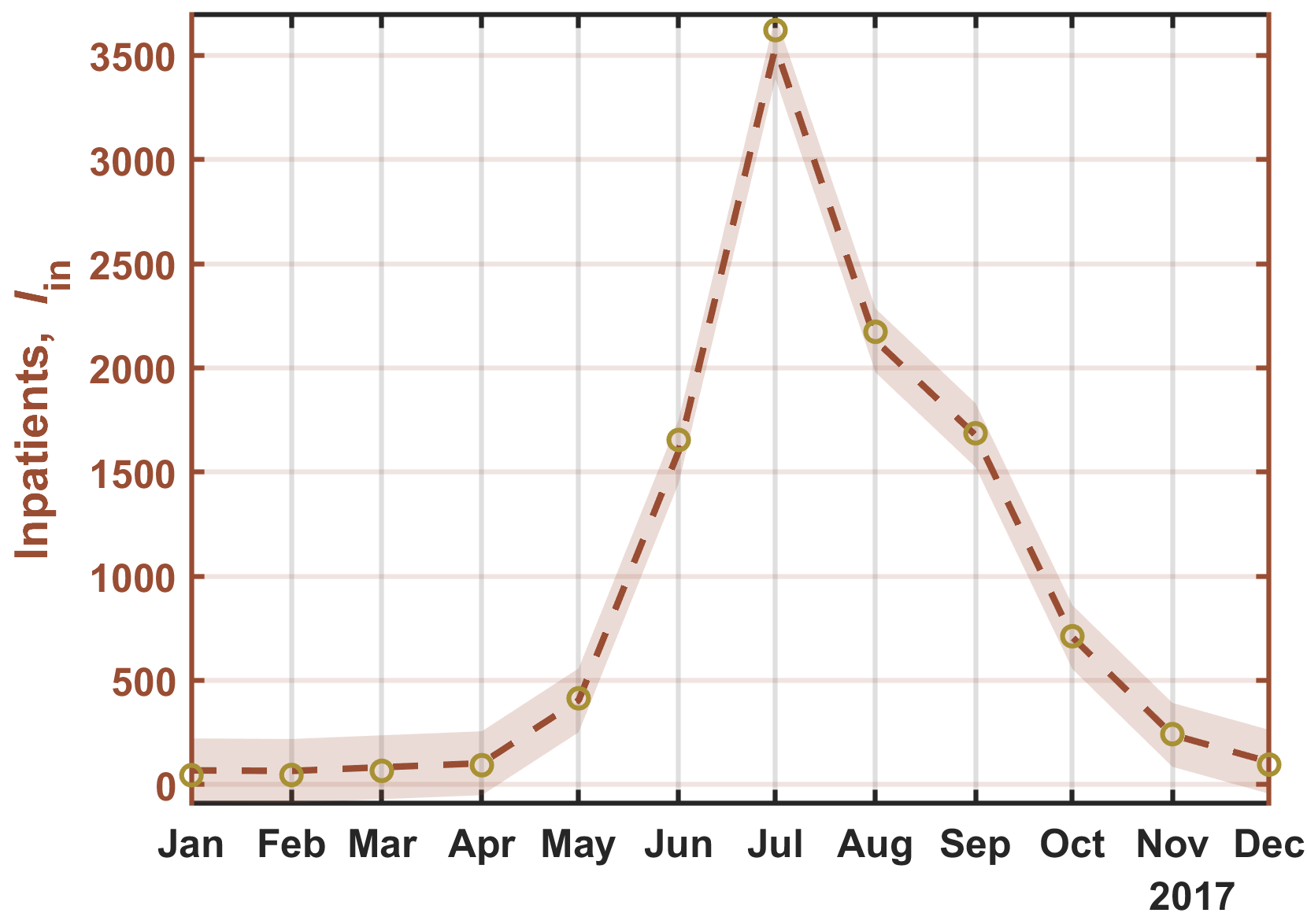


**c)**

## **Fig J. Real-time estimation and forecasting results of HFMD inpatients and outpatients in 2017.** Real-time estimation and forecasting of HFMD inpatients and outpatients in 2017, consistent with Fig E.


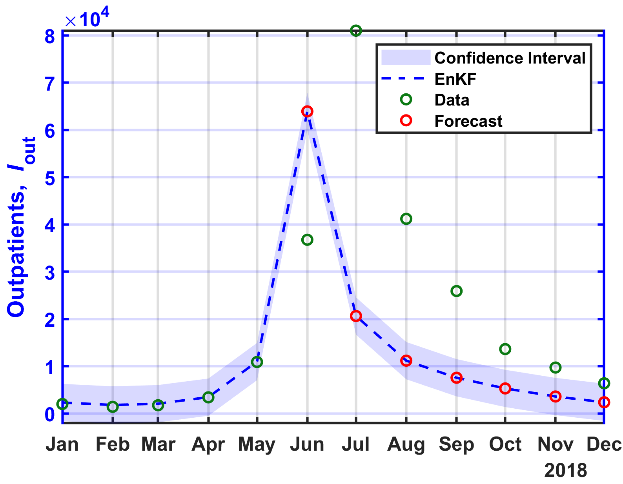

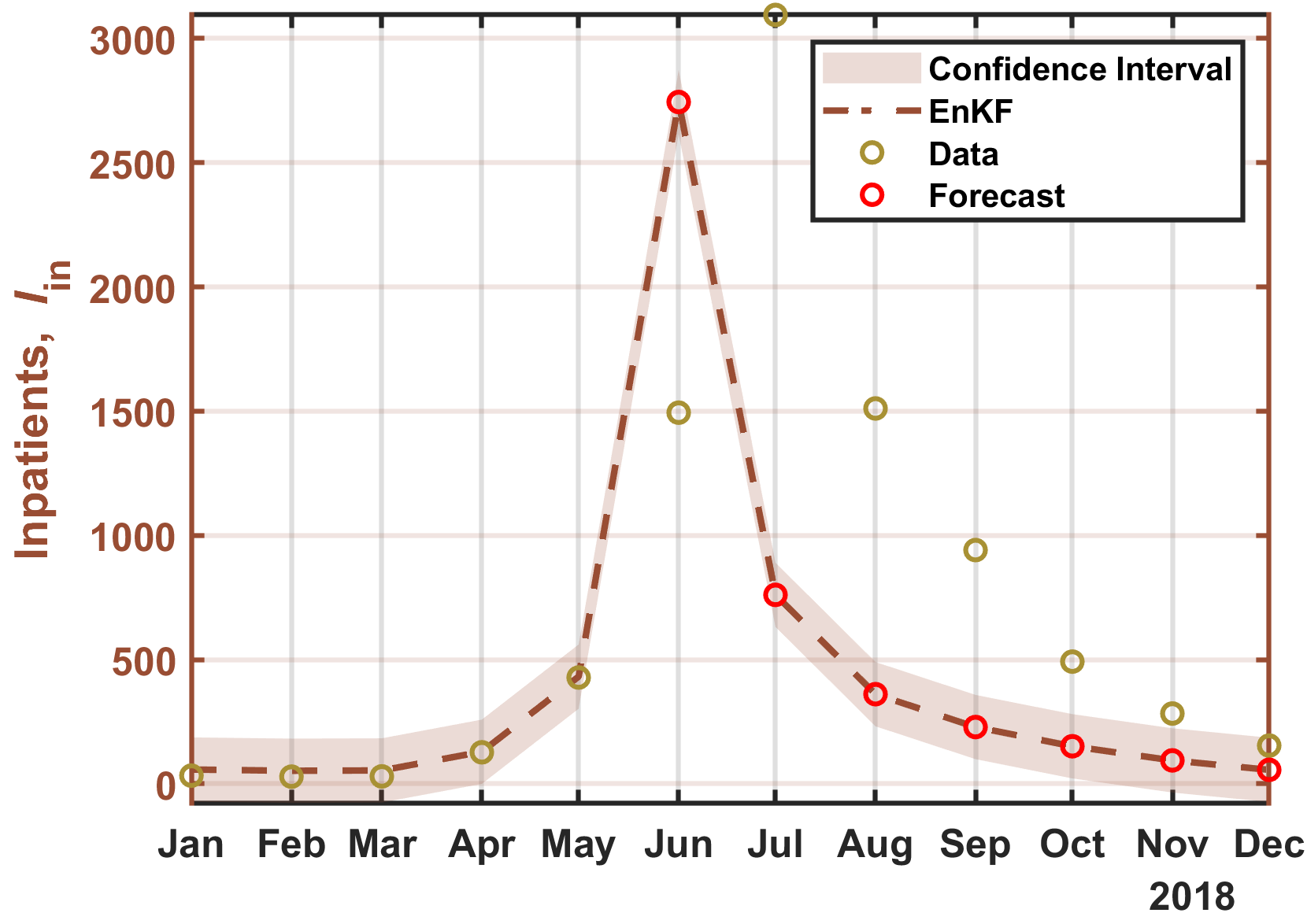


**a)**


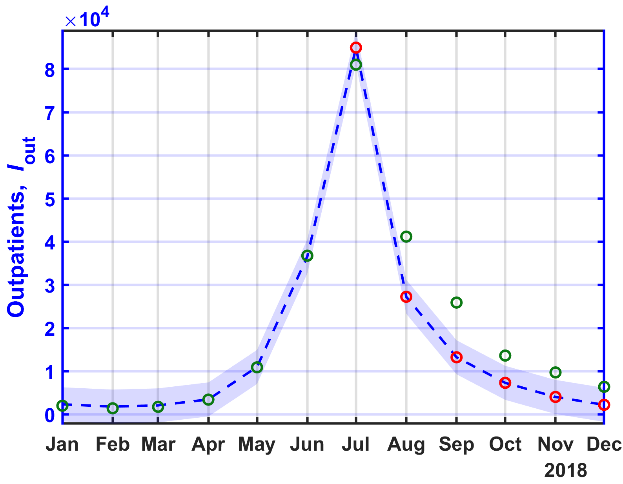

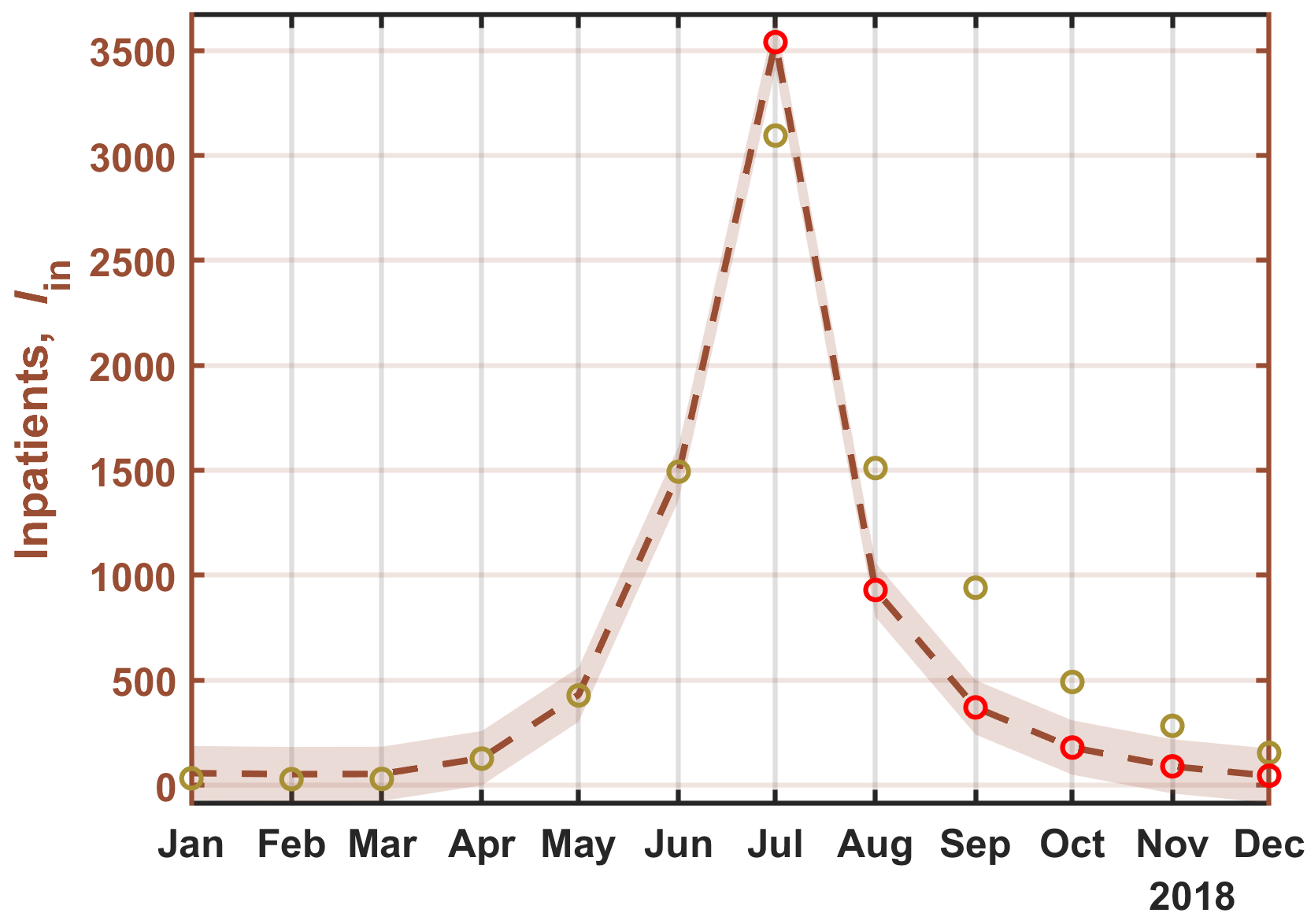


**b)**


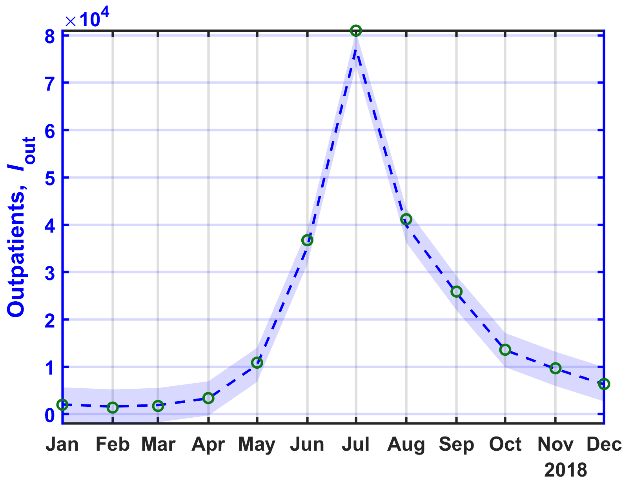

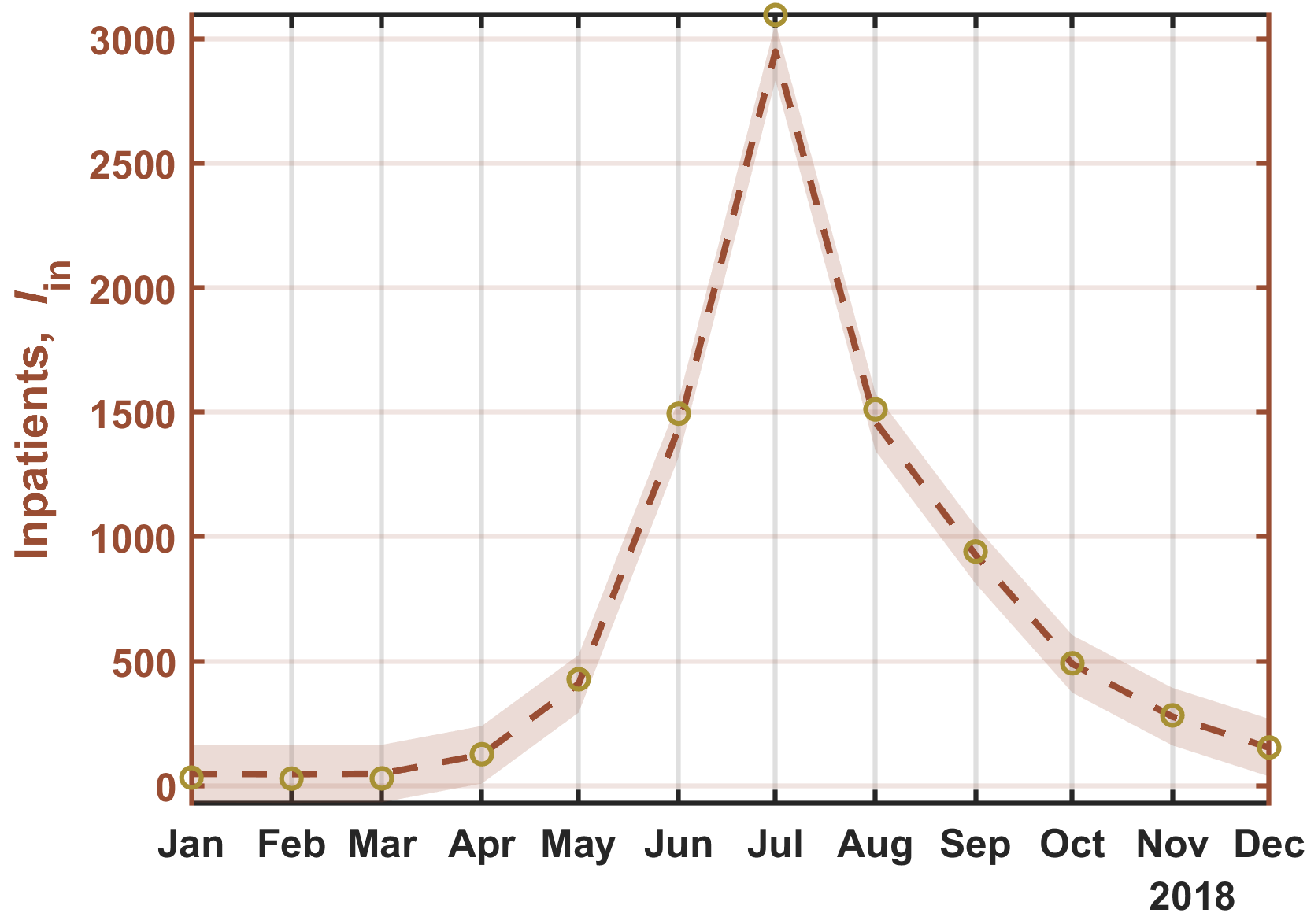


**c)**

## **Fig K. Real-time estimation and forecasting results of HFMD inpatients and outpatients in 2018.** Real-time estimation and forecasting of HFMD inpatients and outpatients in 2018, following Fig E.

**Regression Analysis and Accuracy Assessment**

To further evaluate the discrepancies, regression analyzes were conducted between the observed data and the data predicted from both the prior forecast and the posterior analysis. With an observation error of 10%, the coefficients and R² values of the prior and posterior analyzes showed that the posterior analysis displayed a considerably superior fit to the observed data compared to the prior analysis. We also computed the coefficients and R² values with observation errors of 20%, 30%, 40%, and 50%, and found that the best values occurred at 10% observation error.

Our study involved conducting real-time forecasting from 2011 to 2019, as shown in Figs E-K. We evaluated the accuracy of the peak magnitude forecast by comparing it to the observed peak magnitudes, as illustrated in Fig 3. The data regarding inpatients, outpatients, and the incidence rate for HFMD was accessible. We commenced the process of forecasting the preceding 7 months by utilizing a 5-month dataset, subsequently augmenting the dataset while concurrently reducing the forecast duration. The accuracy assessment during the study period revealed that the forecast for the peak month magnitude was more precise when a greater amount of data was accessible.


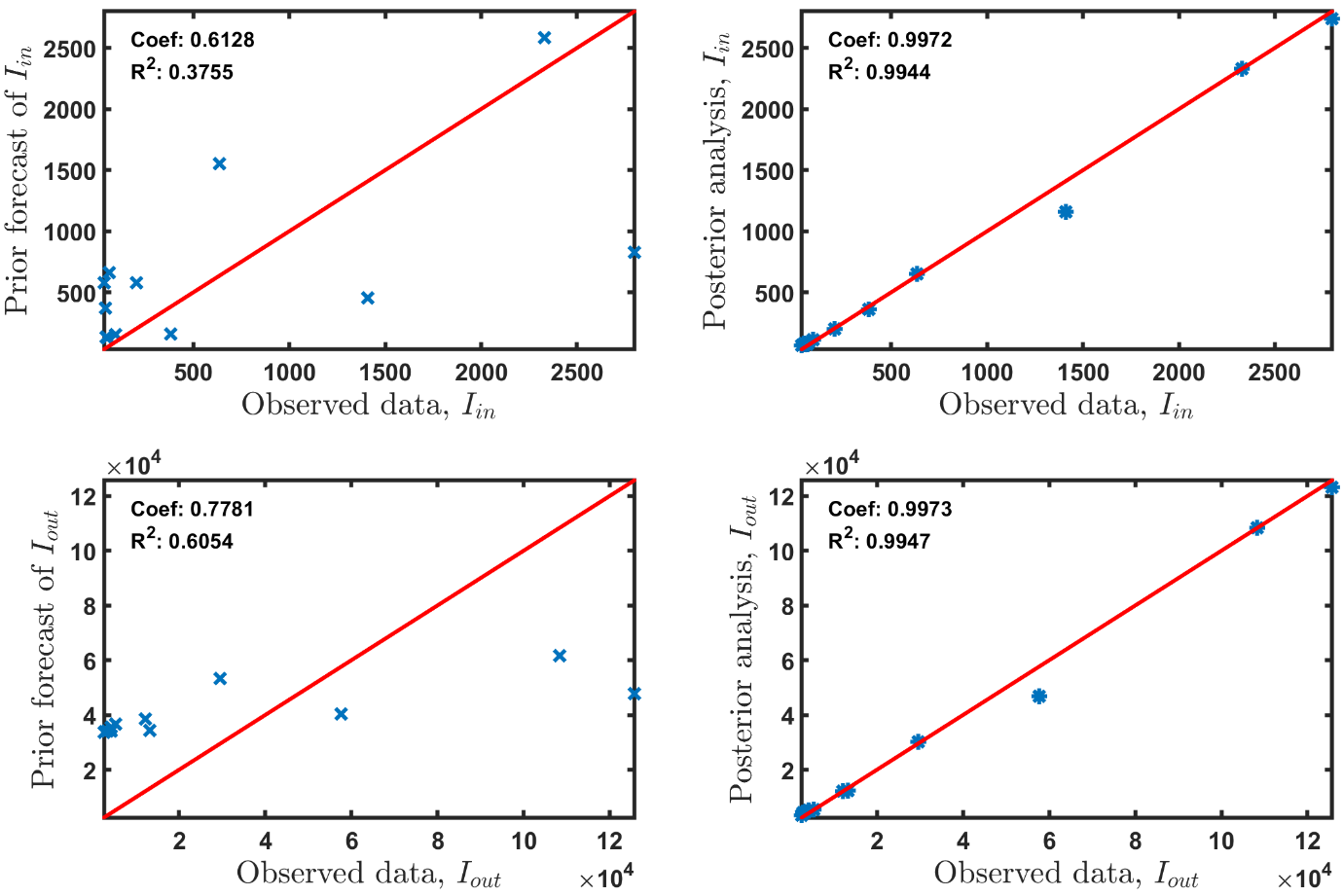


a)


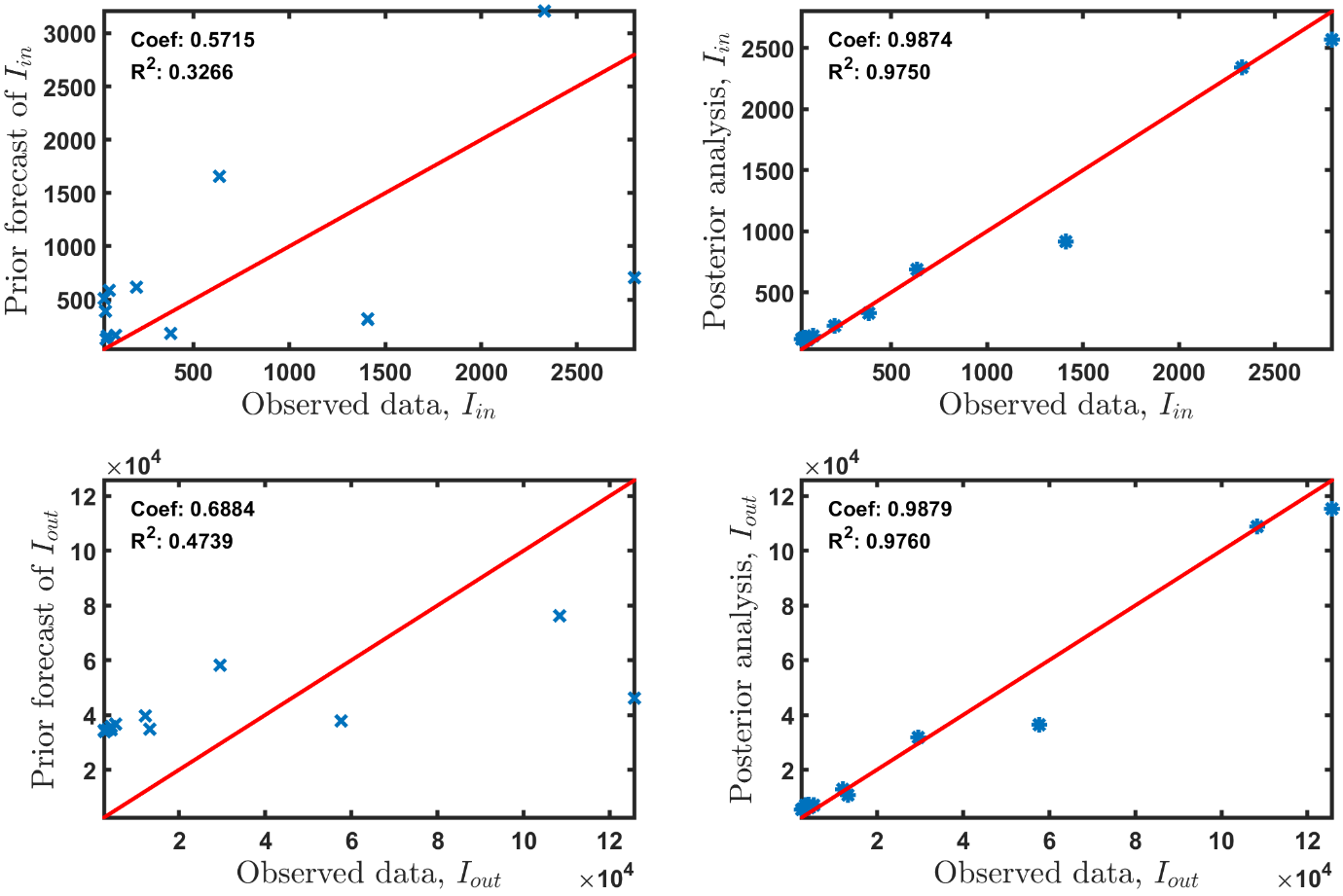


b)


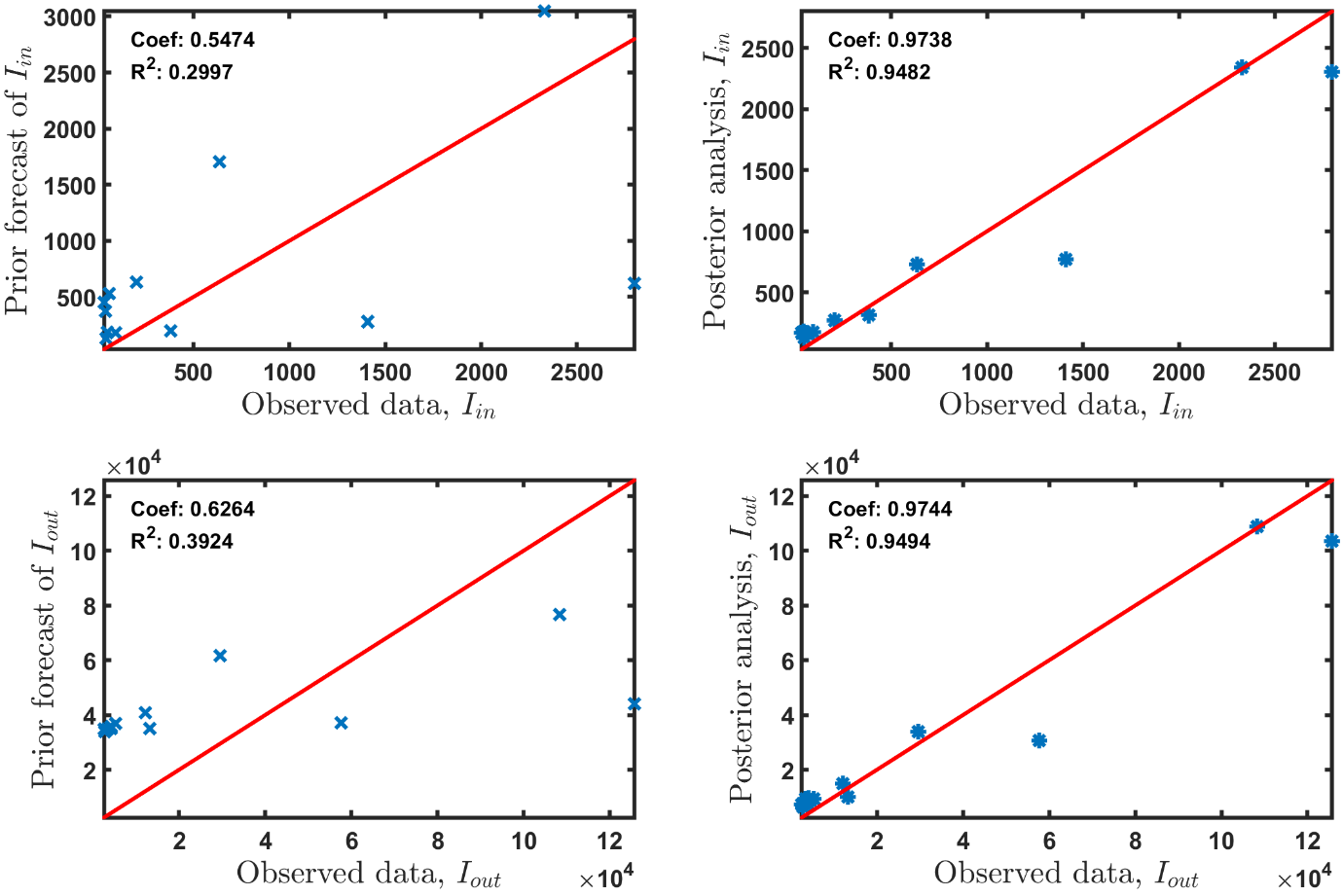


c)


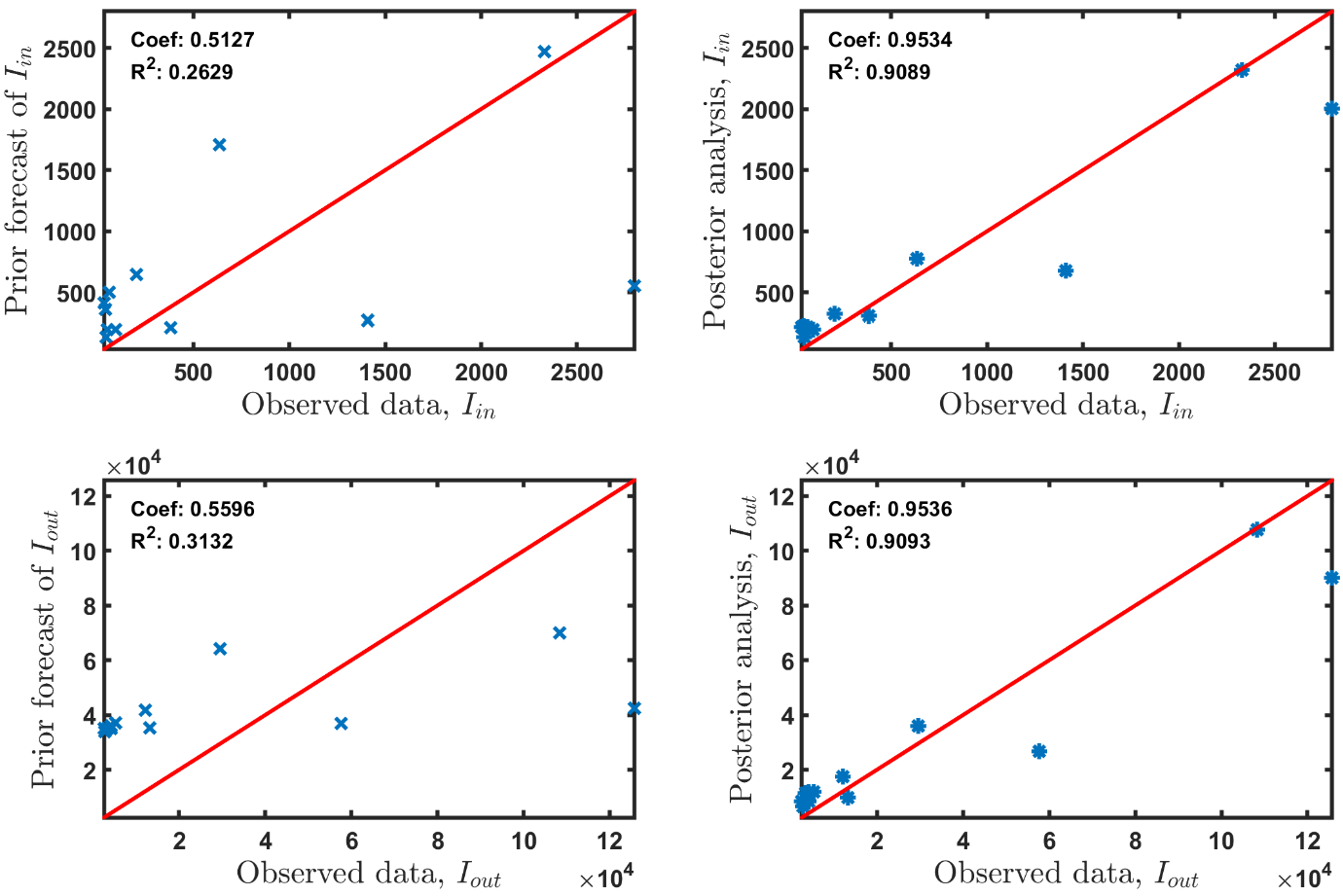


d)


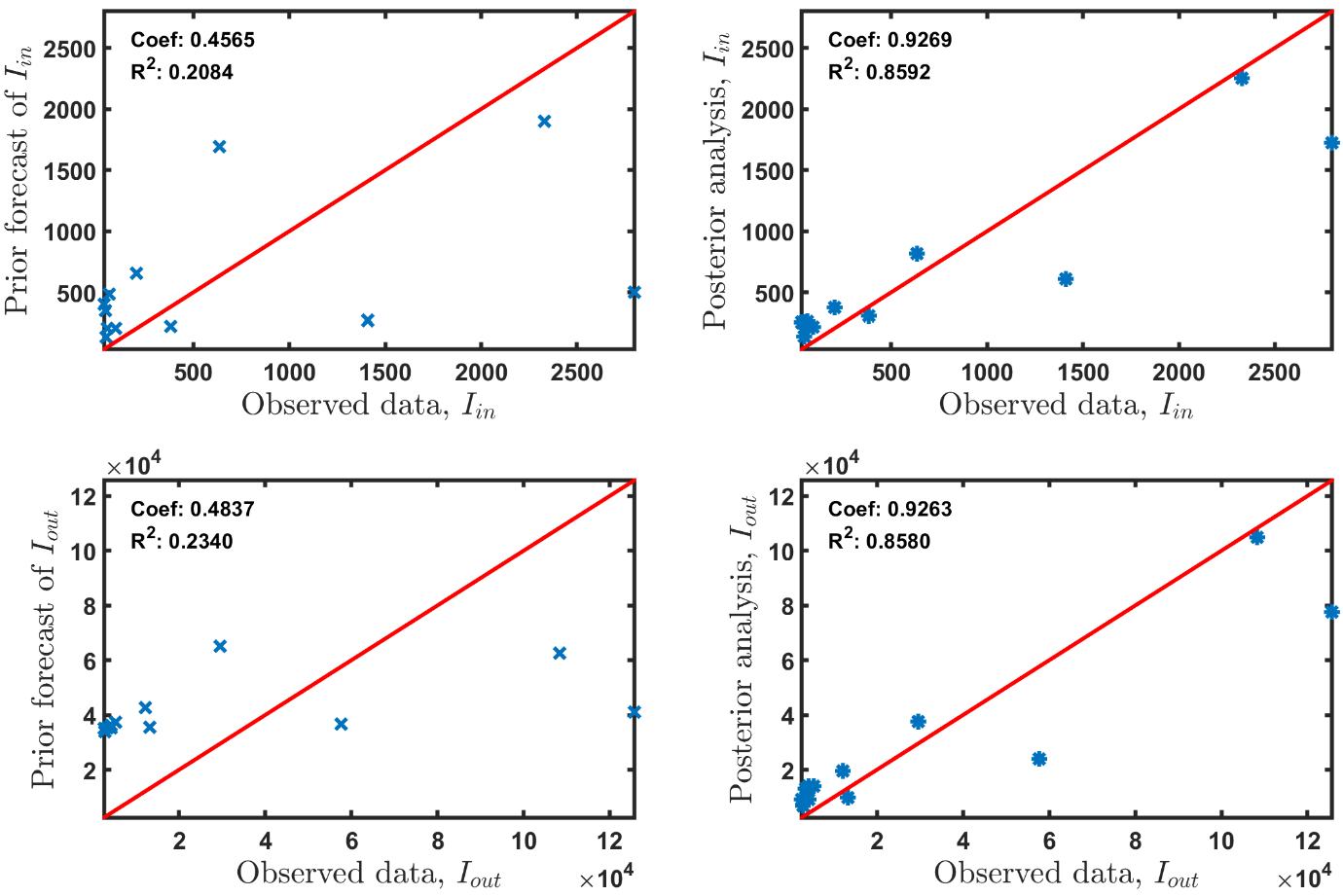


e)

## **Fig L. Prior and posterior analysis of EnKF with different observation errors.** Correlation between observed HFMD data and prior forecasts (left panel) versus posterior analysis (right panel) under observation errors of 10%, 20%, 30%, 40%, and 50%, with coefficients and $R^{2}$ values.
